# Applications of mathematical modelling for assessing microplastic transport and fate in water environments: a comparative review

**DOI:** 10.1007/s10661-024-12731-x

**Published:** 2024-06-27

**Authors:** Tyrone Moodley, Taher Abunama, Sheena Kumari, Dennis Amoah, Mohammed Seyam

**Affiliations:** 1 Department of Civil Engineering and Geomatics, Durban University of Technology, Durban, 4001 South Africa; 2 Institute for Water and Wastewater Technology, Durban University of Technology, Durban, 4001 South Africa; 3 Research Center for Treatment and Management of Water (CEBEDEAU), 4031 Liege, Belgium; 4 Department of Environmental Science, University of Arizona, Tucson, 85721 USA

**Keywords:** Microplastics modelling, Fate and transport, Particle tracking, Hydrodynamic, Process-based, Machine learning

## Abstract

Microplastics in the environment are considered complex pollutants as they are chemical and corrosive-resistant, non-biodegradable and ubiquitous. These microplastics may act as vectors for the dissemination of other pollutants and the transmission of microorganisms into the water environment. The currently available literature reviews focus on analysing the occurrence, environmental effects and methods of microplastic detection, however lacking a wide-scale systematic review and classification of the mathematical microplastic modelling applications. Thus, the current review provides a global overview of the modelling methodologies used for microplastic transport and fate in water environments. This review consolidates, classifies and analyses the methods, model inputs and results of 61 microplastic modelling studies in the last decade (2012–2022). It thoroughly discusses their strengths, weaknesses and common gaps in their modelling framework. Five main modelling types were classified as follows: hydrodynamic, process-based, statistical, mass-balance and machine learning models. Further, categorisations based on the water environments, location and published year of these applications were also adopted. It is concluded that addressed modelling types resulted in relatively reliable outcomes, yet each modelling framework has its strengths and weaknesses. However, common issues were found such as inputs being unrealistically assumed, especially biological processes, and the lack of sufficient field data for model calibration and validation. For future research, it is recommended to incorporate macroplastics’ degradation rates, particles of different shapes and sizes and vertical mixing due to biofouling and turbulent conditions and also more experimental data to obtain precise model inputs and standardised sampling methods for surface and column waters.

**Supplementary Information:**

The online version contains supplementary material available at 10.1007/s10661-024-12731-x.

## Introduction

Plastic accretion in the environment is becoming a significant problem owing to its exponentially expanding manufacturing rate (Boyle & Örmeci, 2020; Eriksen et al., 2014). Model estimates indicate that more than 5 trillion plastic particles float on the ocean surface, and approximately 348 million tonnes of plastic were produced worldwide in 2017 compared to 1.5 million tonnes in 1950 (Boyle & Örmeci, 2020). It is estimated that 75% of the oceans’ waste is plastic, with the majority being classed as microplastics (Uzun et al., 2022). Microplastics, distinguished by being smaller than 5 mm in length, are present in all aquatic environments including the ocean, rivers, lakes and aquifers. These microplastics are typically composed of fibres and films and are either polyethylene, polypropylene, polychloride, polystyrene or polyethylene terephthalate (Zhang et al., 2020). The ocean is considered the largest sink of microplastics, of which 98% originate from land-based sources, with riverine runoff being the primary method of transport (Yang et al., 2021). It is estimated that 1.15–2.41 million tonnes of plastic are deposited into the ocean via rivers annually (Pereao et al., 2020). Microplastics enter the aquatic system in two types, primary and secondary microplastics. Primary microplastics are manufactured products and secondary microplastics are the subsequent breakdown of large plastics (macroplastics) existing in the environment (Boyle & Örmeci, 2020). The main sources of microplastics in the freshwater system come from the effluent of wastewater treatment plants, sewer overflows, runoff from agricultural and industrial sludge and urban and informal settlements (Verster & Bouwman, 2020). Rivers play a crucial role in the transport and fate of microplastics as they operate as conduits for marine plastics, and they transform plastics from primary to secondary microplastics (Verster & Bouwman, 2020). Microplastic transport from rivers to the ocean is reliant on the particle’s specific gravity, heteroaggregation, biofouling, water currents and its interaction with biota. The specific gravity of microplastics may be altered by microbial activity as they attract invertebrates and algae which significantly influence their mobility (Boyle & Örmeci, 2020). When microplastics mix with organic aggregates, biofilm tends to grow on the surface of microplastics owing to its large surface area and polarity, further increasing the settling rate which decreases the buoyancy (Zhang et al., 2020). However, microplastics alter the mechanical structure of sediment, and due to plastic having a low density, the intermolecular area of inorganic minerals becomes larger, and intermolecular forces are destroyed resulting in the sediments being easier to resuspend under the critical shear velocity of 0.67–1.33 cm/s (Zhang et al., 2020). Microplastics are considered complex pollutants since they are chemical and corrosive-resistant, non-biodegradable and ubiquitous in water environments. Microplastics may act as vectors for the dissemination of other pollutants and the transmission of microorganisms into the water environment. The association of microorganisms with microplastics has been reported to boost gene exchange across associated biofilm, promoting the transmission of pathogenic and antibiotic-resistance genes (Eriksen et al., 2014). As the number of plastic waste increases in water bodies, the amount of solar thermal energy inside the water body reduces resulting in the dissipation of surplus energy to the nearby environment (Iroegbu et al., 2020). Microplastics may be broken down by abiotic and biotic mechanisms of degradation when only morphological changes occur. The molecular bonds of plastic are affected by photo, thermal, oxidative, and hydrolytic degradation, of which photodegradation causes the greatest damage. Ultraviolet radiation (290–400 nm) and visible light (400–700) produce electron activity inside the bonds which causes oxidation and cleavage (Boyle & Örmeci, 2020).

Even though microplastic contamination is a trending global issue, many studies have been limited with attention solely focused on coastal water bodies with approximately 4% of studies having a freshwater context, suggesting that rivers, lakes and dams are transport routes to the oceans (Iroegbu et al., 2020; Umlauf, 2019; Wagner & Lambert, 2018). A comment by Haward (2018) states that international initiatives addressing plastic pollution still need to be supported by scientific research, business and community organisations and committed government action that addresses the use, disposal and management of plastics. Many microplastic review studies agree with the comment as (Boyle & Örmeci, 2020; Iroegbu et al., 2020; Mvovo, 2021; Naidoo & Rajkaran, 2020; Sharma & Chatterjee, 2017) all highlight the knowledge gap regarding microplastic pollution data for conservation management and a need to strengthen microplastic education campaigns.

Recently, there has been a rise in freshwater microplastic research, but little is still understood regarding the fate of microplastics (Pereao et al., 2020). Due to the complexity of assessing microplastics, there is a lack of standardised procedures. Hence, many investigations use ecologically unrealistic concentrations which makes real-world applicability problematic (Steinman et al., 2020). Due to the foregoing knowledge gap, researchers are turning to 3-D modelling as a technique to understand and predict the dissemination and mobility of microplastics in riverine environments (He et al., 2021). Mathematical modelling has the potential to understand microplastic transport, fate and distribution in riverine environments, which is demonstrated in various recently developed modelling approaches. Recent research by Uzun et al. (2022), Waldschläger et al. (2022) and Cai et al. (2023) consolidated, characterised and critically analysed modelling frameworks used in microplastic transport research globally and highlighted that all modelling frameworks resulted in relatively reliable findings, with each having its strengths and weaknesses in simulating microplastic transport and fate. However, the studies do have some gaps as Cai et al. (2023) only consider marine-based studies and conclude that hydrodynamic conditions play a crucial role in microplastic transport and hydrodynamic mechanisms in coastal waters differ significantly from those in the open ocean, which highlights the need to consider the riverine environment when assessing microplastic transport. Landscape-scale riverine modelling of microplastic transport and fate is needed to fill the gap in monitoring data, quantify fluxes to the marine environment and identify environmental distribution processes; however, only a few such models exist which limits the capacity to assess microplastic transport and fate and prioritise microplastic reduction interventions (Norling et al., 2024). Waldschläger et al. (2022) consolidates the modelling framework for only natural sediment-based models and highlights various specific limitations for microplastic modelling use. The recurring and crucial limitation found in the study is that microplastics are diverse and are characterised by different densities, size and shapes, as opposed to natural sediments that have been generalised to be spherical. Uzun et al. (2022) systematically consolidate, characterise and analyse 36 microplastic-based modelling studies; however, they do not consider machine-based learning models. Therefore, this review aims to (1) consolidate and analyse the modelling methodologies and results of existing microplastic modelling studies in the aquatic environment; (2) categorise these models by conducting analysis highlighting the studies environment, aim, model framework, model input and main findings; and (3) identify and discuss the strengths and weaknesses of the microplastic models to identify improvements and future research. Table 1 compares the current microplastic modelling-based review articles, including this review, highlighting their differences and main findings.

**Table 1:** Comparison of current microplastic modelling-based review articles

Authors and publication year	Previous review (title)	Covered processes (model inputs)	Modelling types	Modelling environment	No. of reviewed studies/years	Main finding
Current review		Shear stress/drag coefficient, salinity, temperature, biofouling, aggregation, degradation, fragmentation, mechanical breakdown, advection, diffusion, windage, ocean currents, sedimentation, resuspension and beaching	Hydrodynamic, process-based, statistical, mass-balance and machine learning	Marine, riverine, lakes, estuarine, air, soil and surface water	61 (2012–2022)	The modelling types demonstrate relative reliability in simulating microplastic transport and fate, each with distinct strengths and weaknesses. However, common issues persist, including unrealistic assumptions in inputs, limited field data for calibration and a focus on oceanic rather than inland water bodies, hindering the development of accurate global microplastic databases and comprehensive transport models
(Uzun et al., 2022)	Mathematical modeling of microplastic abundance, distribution, and transport in water environments: a review	Settling, resuspension, aggregation, dissolution, degradation, resuspension, burial, windage, ocean currents, beaching, temperature, salinity, advection, diffusion and algae growth	Hydrodynamic, process-based, statistical and mass-balance	Marine, riverine and lakes	36 articles (2012–2020)	More reliable results are obtained using hybrid methods, especially the coupling of hydrodynamic and process-based models, and hydrodynamics and statistical models
(Waldschlager et al. 2022)	Learning from natural sediments to tackle microplastics challenges: a multidisciplinary perspective	Model inputs not specified, however reviewed studies included hydrological inputs (rainfall and temperature), advection, settling, aggregation, resuspension, ocean currents and salinity	Hydrodynamic, mass-balance and process-based	Marnie, riverine, and dams	19 articles (2001–2021)	The possibilities of numerical simulations of sediment transport have not yet been fully exploited for microplastic research, although basic paradigms from sediment transport modelling exist that can be of use for microplastic modelling
(Cai et al., 2023)	A review of methods for modeling microplastic transport in the marine environments	Settling, resuspension, diffusion, aggregation, degradation, fragmentation, beaching, salinity, advection, windage, shear stress and biofouling	Hydrodynamic and process-based	Marine	29 articles (2014–2022)	Hydrodynamic conditions, microplastics physical properties and biota work simultaneously on the transport of microplastics. The dominant hydrodynamic mechanisms in coastal waters differ significantly from those in the open ocean
(Bigdeli et al., 2022)	Lagrangian modeling of marine microplastics fate and transport: the state of the science	Advection, diffusion, windage, beaching, biofouling and degradation	Hydrodynamic and process-based	Marine	15 articles (4 models)	The reviewed models cannot simulate homo- and heteroaggregation, agglomeration or degradation
(Kooi et al. 2018)	Modeling the fate and transport of plastic debris in freshwaters: review and guidance	Advection, aggregation, biofouling, sedimentation, resuspension, degradation and burial	Mass-balance, hydrodynamic and process-based	Freshwater	7 articles (5 models)	The reviewed models only yield accurate estimates if data on spatial variability in emission intensities are available
(Li et al., 2020)	A review of possible pathways of marine microplastics transport in the ocean	Advection, beaching, settling, resuspension, windage and ocean current	Hydrodynamic, and process-based	Marine	23 articles	The uncertainties involved in microplastic transport that require further study include the role of Stokes drift, the shear stress threshold required to separate the two behaviours of suspension and sedimentation and how to evaluate the effects of biofouling and aggregation on the benthic distribution of microplastics
Khatmullina & Chubarenko, 2019)	Transport of marine microplastic particles: why is it so difficult to predict?	Model inputs not specified, however reviewed studies included settling, resuspension, shear stress, windage, beaching, biofouling, temperature, salinity, advection and diffusion	Hydrodynamic, and process-based	Marine	34 articles	The reviewed models reproduce accumulation of floating microplastics however features that need to be addressed are heterogeneous composition of microplastics being their size, density, shape and their evolution depending on the environment

## Review methodology

**Fig. 1:**
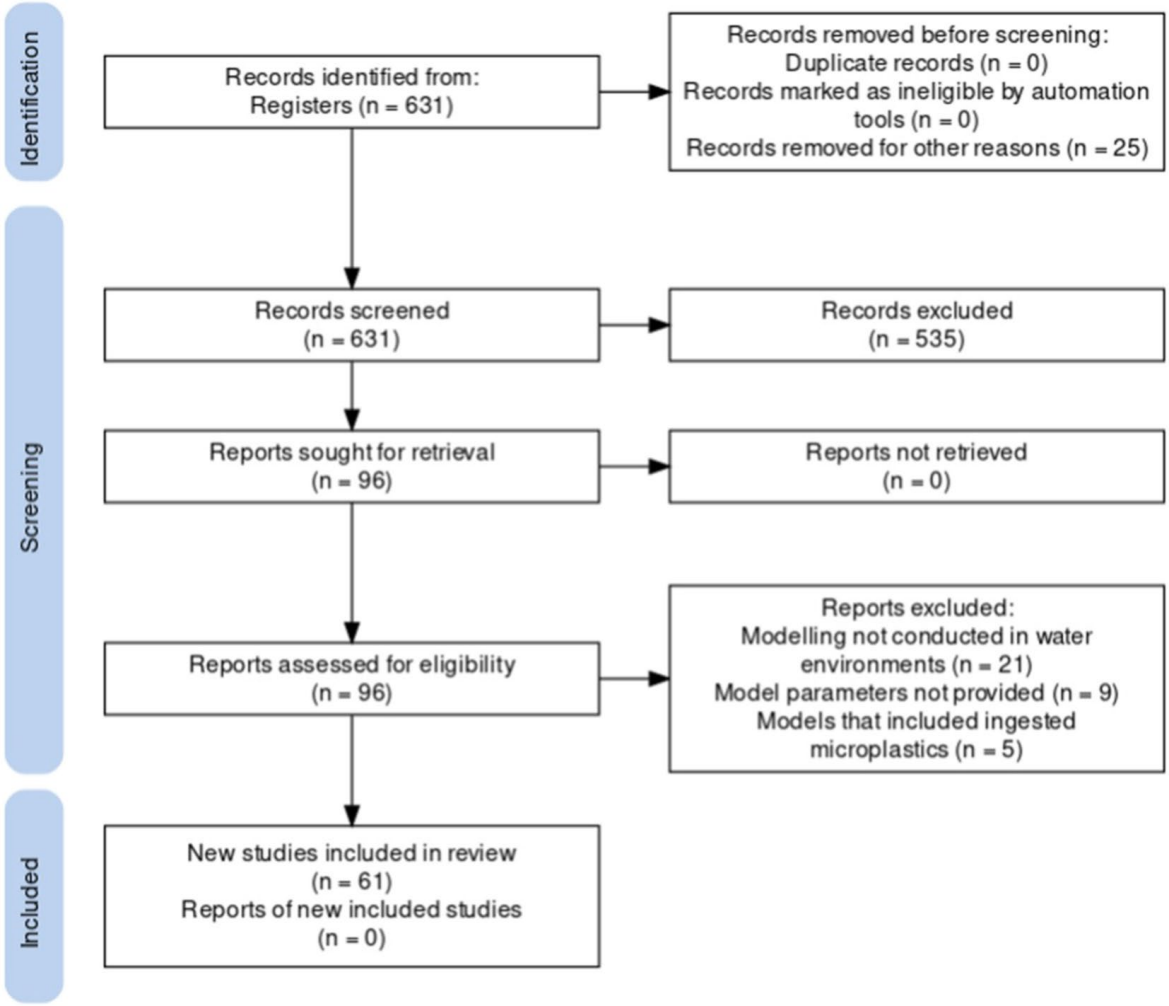
PRISMA flow diagram (created on PRISMA)

**Fig. 2:**
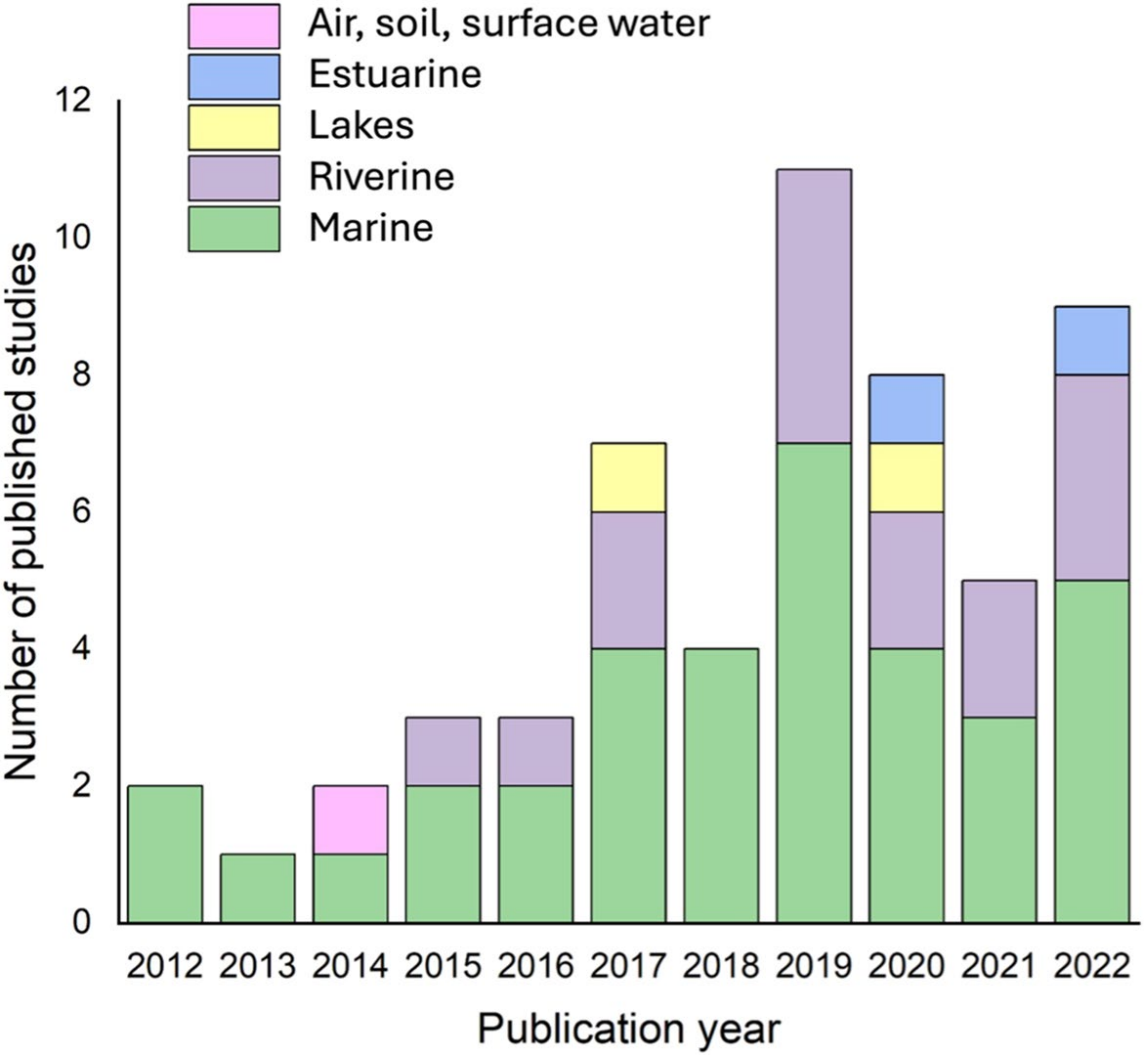
Number of studies on microplastic modelling for various water environments, published in the last decade between 2012 and 2022 (created on SPSS)

A systematic review of microplastic modelling articles concentrating on the identification, transport and fate in water environments was performed to identify the standards of utilised models and to identify future improvements. The review was conducted using the steps of the Preferred Reporting Items for Systematic Reviews and Meta-Analyses (PRISMA (Page et al., 2021)). The data was acquired from Scopus using the query string, “TITLE-ABS-KEY ((microplastic* OR micro-plastic*) AND (model*) AND (transport OR fate) AND (machine learning OR deep learning)”, and Google Scholar to identify relevant publications. It must be emphasised that not all studies relevant to microplastic identification, transport and fate modelling were included in this review as the search was further refined to only include English articles published from 2012, which resulted in 631 articles. The authors’ names, title, year published, abstract and keywords of the identified articles were exported to Microsoft Excel and screened, and the articles that were not centred around microplastic identification, transport and fate modelling were discarded which resulted in 78 articles remaining. The remaining articles were then assessed for eligibility by screening the full text. The studies that were not performed in water environments, included microplastics in fish and provided minimal information and review articles were not considered. Sixty-one scientific articles published between 2012 and 2022, relevant to the modelling of microplastics in aquatic settings, were considered. The study selection process has been summarised in Fig. 1. It was determined that the majority of the research was related to microplastic modelling in the marine environment, with minimal studies available for the freshwater environment. Out of the 61 examined articles, 35 were conducted for the marine environment, 16 for the riverine environment, 4 in lakes, 2 in estuaries, and 1 was conducted for air, soil and surface water runoff, as illustrated in Fig. 2.

The present paper analyses 40 different modelling frameworks under the different modelling classification types, which fills the gap of guiding researchers and professionals towards identifying the correct model, model type and model parameters in accessing microplastic transport and fate in water environments.

The 61 identified articles are presented in supplementary material Table S1 including the author, modelling environment, location, aim/objectives, applied model, model type, model input data and main findings. The “Model classification” section categorises the articles by the type of modelling. The “Microplastic modelling types” section analyses the articles per model classification type and gives an overview of the model application in modelling microplastic transport and fate. The “Applied model comparison” section compares the different models and highlights their strengths and weaknesses, and finally, the “Conclusions and future recommendations” section provides conclusive remarks.

## Model classification

**Fig. 3:**
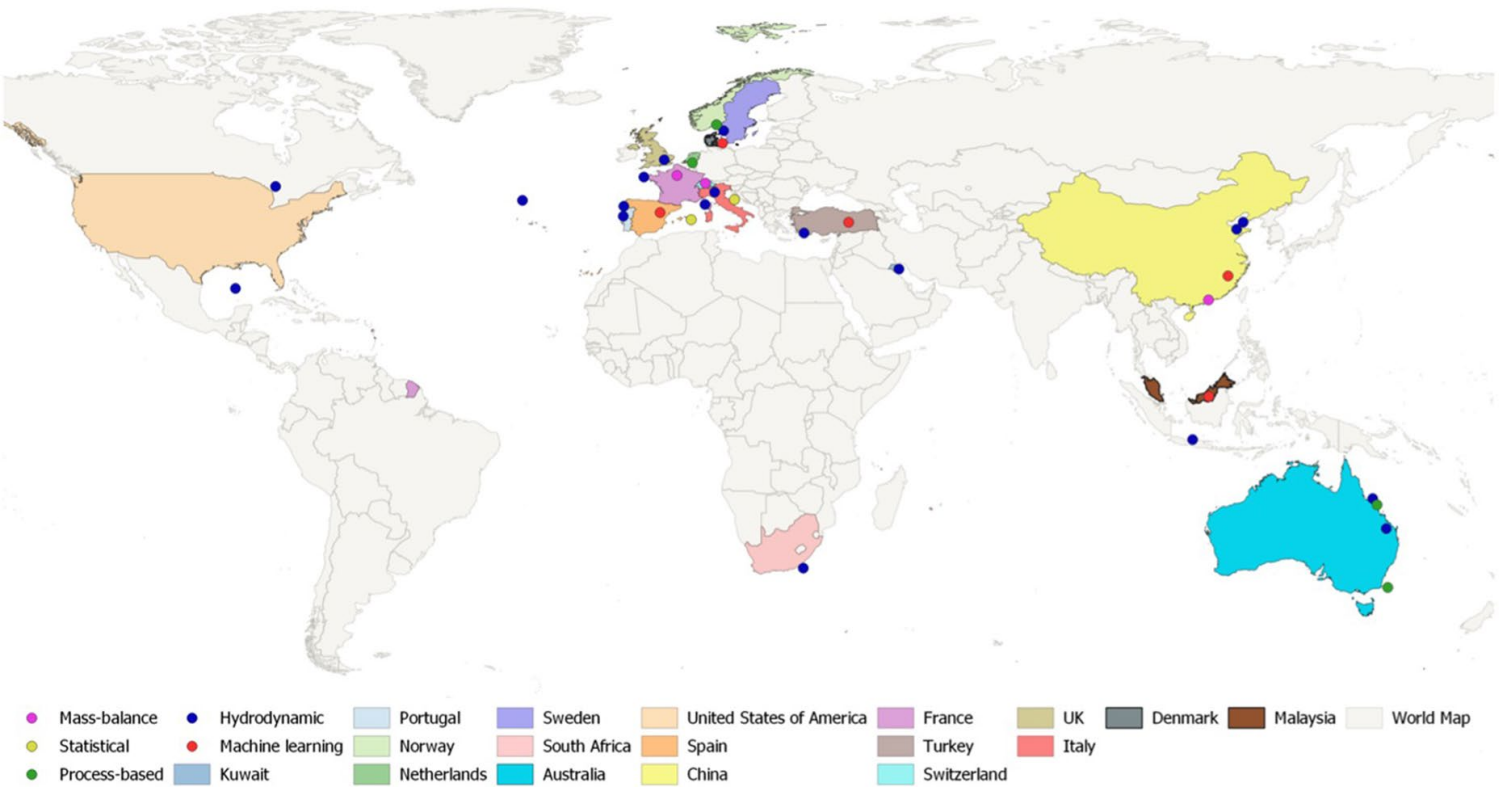
Location of the reviewed studies and modelling types (created on ArcGIS 10.8)

The reviewed articles were categorised by the type of modelling rather than the modelling environment due to most of the examined studies being conducted in the marine (60%) and riverine (28%) environments and a minute proportion being conducted in lakes, estuaries, air, soil and surface runoff (12%). The graphical distribution of the studies and their modelling type are depicted in Fig. 3 which highlights the scarcity and unequal distribution of microplastic modelling research globally, with the majority being conducted in the Northern Hemisphere, Europe.

The models were categorised by either being hydrodynamic, process-based, statistical, mass-balance or machine learning. For this review, hydrodynamic modelling is the modelling of microplastics based on fluid motion created by forces acting alone or in combination, such as tide, wind, waves, gradient and water convergence (Garcia-Rosa et al., 2015). Process-based modelling is characterised as models that address the biological and physical impacts on microplastics such as settling, resuspension, degradation, biofouling and diffusivity. Statistical modelling is based on probabilistic hydrodynamic data; these investigations commonly employ a stochastic modelling framework (Whitehead et al., 2021). Mass-balance models use simultaneous mass-balance equations that use inflow, outflow and distribution of concentration or mass (Coelli et al., 2007). Machine learning models extract correlations from data, to achieve rapid predictions of unknown situations, providing a solution for identifying emerging contaminants (Yu & Hu, 2022).

## Microplastic modelling types

Sixty-one articles, presented in Supplementary material Table S1, are reviewed and discussed based on the above-categorised model type. The articles were also analysed to identify the critical model inputs utilised for the identification, transport and fate modelling of microplastics, being their physical properties, transformational processes and physical transport processes, as tabulated as a checklist in Supplementary material Table S2. The checklist consolidates the model parameters which enables ease of model identification for future research. The physical properties of microplastics, being their size, shape and density, are categorised as input parameters based on the inclusion of shear stress/drag coefficient and salinity as they increase model complexity and competency. The transformation processes are categorised based on the inclusion of biofouling, degradation/fragmentation/mechanical breakdown and aggregation. The physical transport processes are categorised based on the inclusion of advection, diffusion, windage, resuspension/sedimentation and beaching.

### Hydrodynamic modelling

This section analyses 27 hydrodynamic modelling-based studies aiming to consolidate, assess and compare information, model parameters and findings of globally deployed microplastic transport and fate models based on the fluid motion induced by forces.

The analysis explores the efficacy of different modelling approaches in understanding the transport and fate of microplastics in aquatic environments. Three studies focus on comparing 2-D and 3-D modelling techniques which employ various methodologies and models to assess the dynamics of microplastics. While each study contributes valuable insights, collectively they highlight the complexities involved in accurately predicting microplastic transport and fate.

The study by Jalón-Rojas et al. (2019a) highlighted the limitations of 2-D modelling in modelling the complex dynamics of microplastic transport. By comparing 2-D and 3-D modelling approaches, the authors demonstrated that while 2-D modelling can provide general trends, it may lead to inaccuracies, particularly in regions with significant vertical turbulence. This finding emphasises the necessity of incorporating vertical dynamics into models to improve accuracy, especially in areas characterised by strong vertical currents.

Daily and Hoffman (2020) further supported 3-D modelling over 2-D modelling by incorporating sinking velocity and turbulent diffusion into their Lagrangian transport model. Their study highlighted the importance of mixing processes in distributing microplastics more evenly throughout the water column. This finding suggests that without considering mixing, density-driven settling could dominate microplastic movement, potentially leading to biased estimations of pollution distribution.

In contrast, Atwood et al. (2019) presented a comparative analysis between 3-D modelling and remote sensing-based approaches for estimating microplastic accumulation in coastal waters. While the 3-D model incorporated detailed physical forcing and hydrodynamic structure, the remote sensing model relied on imagery analysis to map out the spread of microplastic pollution from river plumes. The study reveals discrepancies between modelled and observed data, attributed partly to the omission of various factors such as windage, microplastic ageing and biofouling in the modelling process.

Despite this limitation, the remote sensing model proved valuable in identifying sources of microplastic outflow, particularly from river mouths. The three studies highlight the importance of adopting sophisticated modelling techniques, particularly 3-D approaches, in accurately assessing the transport and fate of microplastics. While each study contributes valuable insights, they also highlight the need for integrating additional factors such as windage, microplastic ageing and biofouling into models which could enhance their predictive capabilities.

The studies by Collins and Hermes (2019), Frere et al. (2017) and Hoffman and Hittinger (2017) employ the Ichthyop model and offer unique insights into the dynamics of microplastic transport in different aquatic environments, highlighting the significance of hydrodynamic processes. Collins and Hermes (2019) utilised the model coupled with velocity fields, temperature and salinity data. While their study provided valuable initial understanding, it simplifies shoreline interaction by deeming particles beached upon encountering the shoreline. This oversimplification neglects the interaction between hydrodynamic processes such as wind, water currents and thermal gradients, which significantly influence plastic transport and retention. Consequently, the study may underestimate the complexity of plastic pollution dynamics in coastal regions.

Frere et al. (2017) extended the application of the Ichthyop model to examine surface microplastic distribution in the Bay of Brest, France. Their study revealed a transitional convergence zone in the centre of the bay where floating debris tends to accumulate, regardless of hydrodynamic conditions. By incorporating velocity fields from ocean simulations and various atmospheric parameters, the study provided valuable insights into microplastic distribution patterns in coastal areas. However, the reliance on surface observations may overlook the vertical distribution of microplastics, potentially limiting the comprehensiveness of the findings.

In contrast, Hoffman and Hittinger (2017) focused on predicting microplastic transport in lakes using hydrodynamic currents from the Great Lakes Coastal Forecast System (GLCFS) numerical model. Despite not addressing beaching and vertical mixing, their simulated findings align well with in situ data, suggesting realistic model inputs. However, the study’s focus on lakes may not fully capture the complexities of marine microplastic dynamics, potentially limiting its applicability to coastal and oceanic environments.

The following studies investigate sedimentation dynamics of microplastics, highlighting the complex relationship of factors such as particle size, density, buoyancy and hydrodynamic processes. The study by Daily and Hoffman (2020) emphasised the importance of mixing in mitigating the dominance of density-driven settling over advection in microplastic movement. This finding contradicts earlier results by Enders et al. (2015), who demonstrated that small microplastics (< 200 μm) exhibit horizontal dispersal patterns due to fragmentation and reduced residence time in the ocean surface layer. Bondelind et al. (2020) further supported the idea of horizontal flow for minute low-density microplastics, particularly in the context of tyre wear particles in stormwater runoff. Their use of 3-D modelling with MIKE 3 FM software models the settling behaviour of different-sized particles, emphasising the non-settlement of minute microplastics. Similarly, Nizzetto et al. (2016) highlighted the importance of particle size rather than density in sedimentation, suggesting that microplastics of 0.2 mm are not retained in river sediments regardless of density.

Mountford and Morales Maqueda (2019) introduced the significance of buoyancy in microplastic sedimentation, particularly in coastal and benthic areas. Their study, utilising a 3-D ocean model, highlighted the importance of incorporating biological and physical–chemical processes such as biofouling and degradation to enhance model realism. This highlights the complexity of microplastic dynamics and the limitations of purely hydrodynamic models. He et al. (2021) further explored the transport processes of sedimental microplastics, highlighting the role of particle density, size and shape, among other factors. Their utilisation of a 3-D particle transport model offers detailed insights into microplastic dispersal and deposition, complementing previous studies’ findings on particle behaviour.

The following studies by Alosairi et al. (2020), Diez-Minguito et al. (2020) and Critchell et al. (2015) each employed different modelling approaches to investigate microplastic behaviour, focusing on hydrodynamic conditions and windage. Alosairi et al. (2020) utilised the Delft3D modelling framework to study microplastic transport and fate under dominant wind conditions and reverse estuarine circulation. Their study demonstrated the significant influence of various physical characteristics, including particle size, density, release time, diffusivity and windage, on microplastic mobility. Notably, windage emerged as a crucial factor affecting microplastic behaviour. However, the analysis also highlights the importance of considering additional factors beyond windage, as demonstrated by the findings of other studies.

Diez-Minguito et al. (2020) and Critchell et al. (2015) both challenged the idea that windage is the most significant factor in microplastic transport. Diez-Minguito et al. (2020) employed a 2-D vertical model to analyse microplastic distribution in an estuary, highlighting the influence of river discharge, wind-driven circulation and density-driven circulation. Their findings suggest that while winds dominate circulation in certain areas, gravitational circulation becomes predominant in others.

Critchell et al. (2015) also highlighted, using the SLIM model, that the orientation of beaches to prevailing wind directions significantly impacts the accumulation rate of plastics. Moreover, they find that while wind drift coefficients and discharge timing minimally affect plastics from rivers, they notably affect debris from ships. This highlights the importance of considering the specific sources and pathways of microplastics in modelling studies.

The following studies employ diverse modelling techniques, including Lagrangian transport models, Regional Ocean Modeling Systems (ROMS), particle tracking modules and experimental approaches.

Zhang et al. (2022) explored an unexpected shoreline microplastic leakage event using the LTRANS and ROMS models, revealing the significant influence of water currents on microplastic transport. By simulating particle transport with LTRANS, they demonstrated the role of irregular coastlines and surface currents in dispersing microplastics over long distances. However, the study’s reliance on modelling approaches may oversimplify the complexity of real-world conditions and environmental interactions, potentially limiting the accuracy of predictions. Similarly, Yu et al. (2018), who also used the ROMS and LTRANS, overlooked environmental processes when simulating factors influencing coastal microplastic distribution, affecting the accuracy of the findings.

Studies by Genc et al. (2020) who employed the HYDROTAM-3D model to study microplastic accumulation emphasised the importance of considering wind, wave and density-driven currents; Handyman et al. (2019) who utilised the MIKE 21 Flow Model FM to analyse microplastic transport and accumulation patterns highlighted the influence of wind and current data on microplastic movement; and Eriksen et al. (2014) and Lebreton et al. (2017) who employed modelling approaches to quantify plastics in the world’s oceans and predict plastic inputs from rivers into oceans resulted in reliable findings highlighting the value of integrated modelling frameworks in determining microplastic transport dynamics. While their studies provide valuable insights, the reliance on model simulations may oversimplify the complexity of environmental and microplastic transport processes and fail to capture uncertainties associated with model inputs and assumptions.

Ding et al. (2019), Ballent et al. (2013) and Cook et al. (2020) employed experimental and modelling approaches to replicate microplastic movement and transport behaviour. While their studies provide valuable experimental insights, the scale and conditions of the experimental setup may not fully replicate real-world environmental conditions, limiting the validity of their findings.

### Process-based modelling

Six articles employed the process-based modelling approach to determine the transport and fate of microplastics taking into account the biological and physical impacts.

The first reviewed process-based study, presented in Table S1, was by Li et al. (2018) who investigated the trapping effect of semi-closed seas on microplastic transport using the SLIM model, highlighting the significance of seasonal fluctuations, source locations and hydrodynamic conditions. Critchell and Lambrechts (2016) and Li et al. (2018) also agreed highlighting the significance of source location and additionally degradation rate, resuspension and settling rate. While the study’s sensitivity analysis yields valuable insights, their reliance on a 2-D modelling approach may oversimplify vertical transport processes, potentially limiting the accuracy of predictions, particularly in deeper waters. However, the validation of results with field data enhances the credibility of findings. However, 3-D modelling could better resolve plastic oceanography in deeper waters as suggested by Jalón-Rojas et al. (2019a) and Daily and Hoffman (2020).

Sani-Kast et al. (2015) investigated the impact of environmental conditions on nanoparticle fate, emphasising the importance of source site circumstances in determining nanoparticle distribution and fate. While the study’s focus on nanoparticles offers valuable insights, its findings may not be directly applicable to larger microplastics due to differences in transport mechanisms and behaviour. Additionally, the reliance on a modified river multimedia box model may overlook the complexity of nanoparticle interactions in real-world river systems as Jalón-Rojas et al. (2019b) conducted a sensitivity analysis using the TrackMPD model explaining the significance of sinking, turbulent dispersion, beaching and biogeochemical factors in shaping microplastic distribution and accumulation; however, the reliance on modelling simulations necessitates accurate parameterisation and validation against field data to ensure the reliability of findings.

In a previously reviewed hydrodynamic study which omitted biological processes, He et al. (2021) indicated that microplastics with low density have high mobility. However, biological inclusion might be one of the main variables that influence the amount and distribution of microplastics in the water column. The work by Berezina et al. (2021) and Besseling et al. (2017) addressed the influence of biogeochemical processes on microplastic transport by integrating biochemical and biogeochemical models. Their findings highlight the role of biota and organic matter in transporting light-density floating microplastics into deep layers and sediments. However, the complexity of model interactions and scenario-based parameterisation may introduce uncertainties in predictions, regarding the representation of complex environmental interactions.

### Statistical modelling

The analysed studies employ statistical models, particularly the Markov chain model, to analyse the transport and fate of microplastics in the Adriatic Sea. They offer valuable insights into the dynamics of plastic debris movement, highlighting their pathways, and accumulation areas. However, the reliance of primarily statistical techniques introduce uncertainties and limit accuracy of predictions, particularly implications inherent in complex hydrodynamic interactions and environmental factors. This was evident in the study by Coppini et al. (2018) and Liubartseva et al. (2016), with their assumptions made in establishing parametric probability functions for beaching and sedimentation processes impacting the accuracy of predicting particle half.

The study by Liubartseva et al. (2018) also relied on simplified parameterisation, including vertical displacement, which affected the reliability of emphasising the crucial influence of coastal area kinematics on microplastic fate. Additionally, Maximenko et al. (2012) adopt a stochastic modelling approach to study marine debris pathways using historical trajectories of drifting buoys. While the study identifies primary areas of drifter aggregation, the reliance on historical data and simplifications in the modelling approach may introduce biases and limitations in the analysis.

### Mass-balance modelling

The analysed studies employed mass-balance models to determine microplastic fluxes and inventory, globally. The studies by Siegfried et al. (2017), Mai et al. (2019), Van Wijnen et al. (2019) aimed at determining microplastic export by rivers highlighting anthropogenic activities. All studies experienced the same limitations of uncertainties in input field data and the lack of a validated model which limited the confidence in forecasting river export.

However, in studies by Unice et al. (2019) who modelled the fate and transport of tyre and road wear particles, Koelmans et al. (2017) who investigated plastic removal processes from the near-surface ocean, and Domercq et al. (2022) who modelled the transport and fate of nano- and microplastics, it was found that even though there were uncertainties in the input data, the model verification against field observations suggests plausibility.

Meesters et al. (2014) was also limited by uncertainties due to a lack of information on emission rates and physicochemical properties, as they modelled nanoplastics, highlighting its utility in environmental risk assessments.

### Machine learning modelling

The utilisation of machine learning (ML) techniques for the identification, classification and prediction of microplastics presents a promising approach to addressing the challenges associated with manual analysis and detection. The studies reviewed demonstrate the effectiveness of various ML algorithms in different contexts, ranging from image classification to spectroscopic analysis.

One of the commonly employed ML techniques is artificial neural networks (ANNs), as demonstrated by Guo and Wang (2021) and Yurtsever and Yurtsever (2019). The high correlation coefficient values obtained in predicting sorption capacity highlight the potential of ANNs in capturing complex relationships between variables (Seyam et al., 2020). Similarly, convolutional neural networks (CNNs) have shown remarkable performance in image classification tasks, achieving high accuracy rates in identifying microplastics from images (Yurtsever & Yurtsever, 2019). However, the effectiveness of CNNs may be influenced by factors such as the choice of architecture and training dataset quality, as evidenced by variations in accuracy rates across studies.

Support vector machines (SVMs) have also emerged as a powerful tool for microplastic identification, offering highly accurate models across different sizes and types of microplastics (Lin et al., 2022). The ability of SVMs to handle limited sample data and provide accurate classification highlights their suitability for microplastic detection tasks. However, the performance of SVMs may be sensitive to parameter tuning and the choice of kernel function, requiring careful optimisation to achieve optimal results (Abunama et al., 2019).

Decision tree (DT) and random forest (RF) algorithms have been applied successfully in microplastic detection and polymer identification tasks, offering high accuracy rates in predicting particle origins and polymer types (Meyers et al., 2022). The interpretability of decision trees makes them particularly useful for understanding the underlying decision-making process. However, the performance of decision tree-based models may degrade with increasing complexity of the dataset and the presence of noisy features.

Spectroscopic techniques coupled with ML classifiers have shown promise in characterising microplastics, with ATR-FTIR identified as the most effective technique for coupled with ML classifiers (Michel et al., 2020). Similarly, hyperspectral imaging combined with partial least squares discriminant analysis (PLS-DA) has demonstrated superior performance in microplastic characterisation compared to other ML models (da Silva et al., 2020).

While the reviewed studies highlight the potential of ML techniques in microplastic detection, classification and prediction, several challenges and limitations remain. The generalisability of ML models may be limited by variations in environmental conditions, sample composition and data quality. Additionally, the interpretability of complex ML models such as deep neural networks may pose challenges in understanding the underlying decision-making process. Furthermore, the lack of standardised protocols and datasets for microplastic analysis may hinder the comparability and reproducibility of results across studies.

## Applied model comparison

**Fig. 4:**
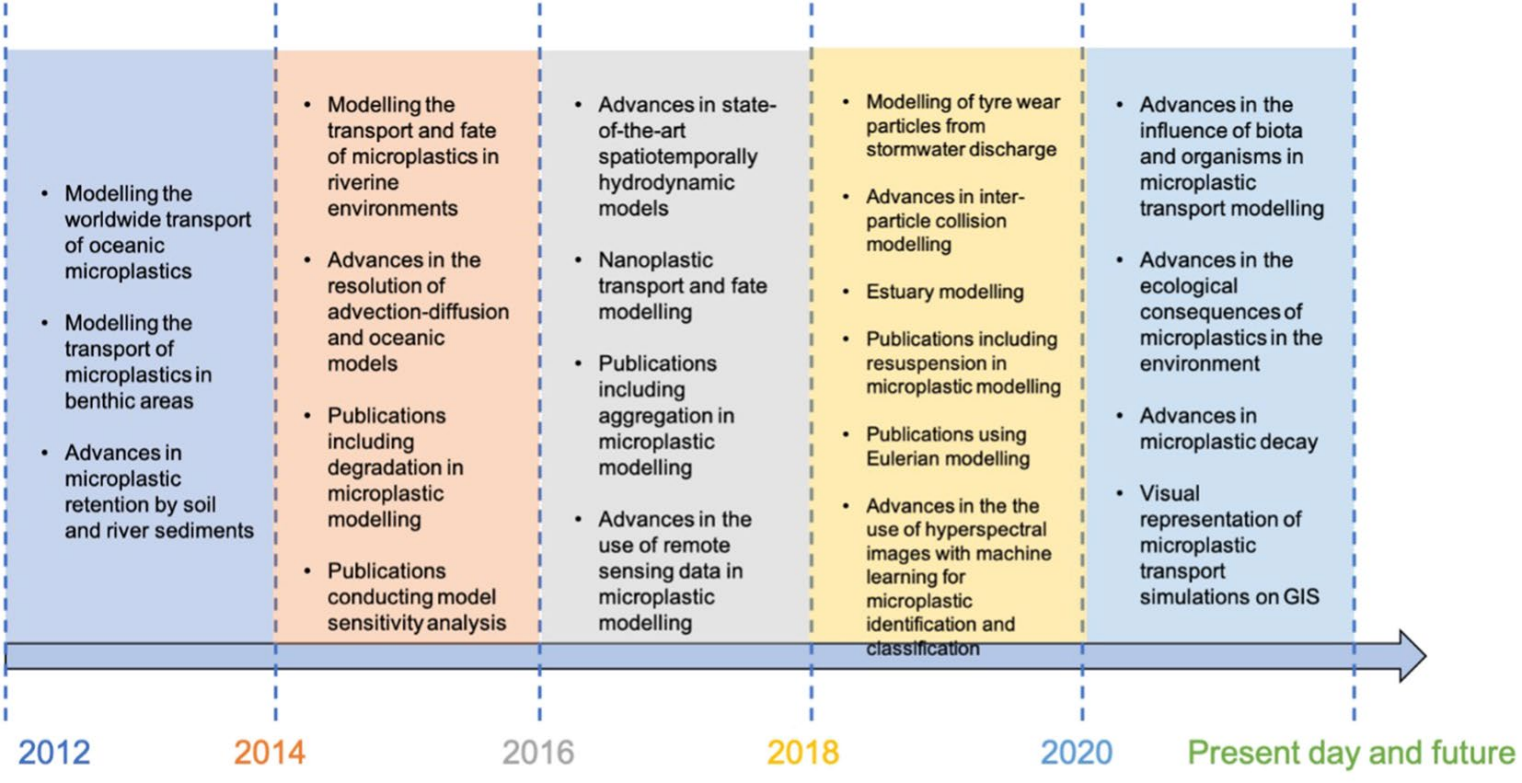
Milestones in the historical evolution of microplastic modelling (time vs microplastic modelling standards (created on Microsoft PowerPoint)

In this review, 61 microplastic modelling articles, acquired from standard scientific online resources, are reviewed and discussed according to categorisation by the dominant modelling framework utilised, which highlighted milestones in the historical evolution of microplastic modelling since 2012 as illustrated in Fig. 4. The discussion is centred on the model employed, the inputs into the model and the model outcomes.

### Hydrodynamic modelling

Hydrodynamic models are adaptable and can be applied in any aquatic condition in which hydrodynamic data is accessible. The computational efficiency of the models can be adjusted depending on the size and resolution necessary, although models can become computationally intensive when simulating vast regions at high resolution. In several studies, hydrodynamic modelling outcomes tend to decline in accuracy as simulation duration increases and resolution drops. However, given the fine-scale resolution owing to models utilising triangular elements, model results demonstrated high conformity with in-situ data.

Sediment interaction is also scarce in hydrodynamic modelling since resuspension is not frequently included. 3-D hydrodynamic modelling offers more accurate results, while 2-D modelling may lead to inaccuracies owing to its single sigma velocity layers. 3-D modelling is advantageous, notably for areas with severe vertical current shear as when mixing is incorporated in the modelling, the plastics are distributed more uniformly throughout the water column. The transport of microplastics resulting from hydrodynamic modelling indicates that microplastic concentration is influenced by the vertical water density gradient induced by saline water and large to medium-sized particles settle, whereas small microplastics tend to not settle, irrespective of their density. Larger microplastics with densities slightly greater than water are maintained in the sediment, while minute microplastics are disseminated horizontally, but their residence time in the surface layer is considerably lower than larger plastic debris.

The results of the hydrodynamic models are affected by many factors. However, sensitivity analysis indicates that particle size, receiving water density, number of particles, release time, diffusivity, water currents and windage significantly affect microplastic behaviour and mobility, with windage having the greatest effect. Globally, there is a lack of microplastic data, the quantity and the factors that affect microplastic movement. To enhance modelling accuracy, incorporate additional factors like land use characteristics, economic activities, transportation, urbanisation, construction, tourism, fishing and aquaculture industries into microplastic models. When crucial elements are omitted from hydrodynamic modelling, viz. windage, microplastic ageing, biofouling, fragmentation and settling, the modelling may not be accurate and result in minimal correlation with sampled data. If process-based modelling parameters were included in the modelling such as degradation and biofouling, it would increase microplastic density, leading to the reduction of buoyancy and eventually sinking, affecting the transport modelling results. However, these biological and physical–chemical processes can be difficult to determine (Table 2).

**Table 2:** Strengths and weaknesses of the reviewed hydrodynamic models

Hydrodynamic Models	Advantages	Disadvantages
The Delft3D-PART	The model can be applied to coastal, river, lake, and estuarine water bodies.	The model would require an extensive computational capacity to consider all release particles.
	The model provides a detailed mathematical solution for time-dependent tidal force, varying wind fields, and density gradients based on temperature and salinity.	
	Resolves particle displacements at sub-grid scales while maintaining a reasonable computational cost.	
Ichthyop	The modelling software is free.	Shoreline interaction is over-simplified.
	The model can continuously track microplastic transportation.	The open boundary allows for the possible entry of particles or re-entry of microplastics.
	The model can upscale point measurements to larger areas.	More input parameters should be available for the vertical movement of microplastics as the process is complex.
		Outputs and sources are not detailed.
		The model is suited for considering the settling velocity of prolate spheroids only.
		Not able to model biofouling and degradation.
MOHID Water Model System	Model is high-resolution.	Model can only be used in marine environments.
	Model can integrate different oceanographic processes.	
MIKE 3 FM	The model can describe local catchments.	Model is computationally expensive.
	Model can simulate large areas at high resolutions.	
SLIM model	Highly versatile as it uses triangular elements.	Model is limited to oceans.
	The model has fine-scale resolution at complex oceanography and coarse scale at open water movements.	The model does not consider the effect of hills on islands and headlands.
	The model has a hydrodynamic component and an advection-diffusion component.	Degradation rates are not considered.
	Model is accurate at a local scale.	
Idealized 2D-vertical model	Model is high-resolution.	Degradation rates are not considered.
	Accurate results.	
Lattice Boltzmann method	Accurate showing good agreement with measured data.	Vertical transport is not considered
	The model treats the fluid as fractional particles in regular lattices for simplification rather than using shallow water equations.	
	Good performance for flow with complex boundary conditions.	
	It has both Eulerian and Lagrangian attributes.	
HYCOM and Pol3DD models	Accurate results.	Velocity in the surface layer is only considered.
Numerical circulation model	Model is high-resolution.	Results are progressively unreliable with increasing simulation time.
HYDROTAM-3D	Model is high-resolution.	Model does not consider resuspension.
	Model is computationally efficient.	
Mike 21 Flow Model	Model is high-resolution.	
TUFLOW FV Particle Tracking Module	Model is high-resolution.	Model is computationally expensive.
	Accurate results.	
	Model can simulate process-based parameters.	
	Model outputs are in the form of maps and files that can be processed on GIS.	
Numerical model (4th order Runge-Kutta) and GLCFS model.	Model is high-resolution.	Propagation of particles is not completed within the model.
	Model is accurate.	The model does not consider inter-lake transport.
The Particle Tracking and Analysis Toolbox	Model is high-resolution.	Particle sinking is not considered.
Global model	Model is accurate for most locations.	The model does not include multiple source scenarios to account for inputs from different sectors.
Pol3DD	Model is high-resolution.	Only velocity in the surface layer is considered.
	Model is accurate.	
Nucleus for European Modelling of the Ocean Version 3.6, configuration ORCA2‐LIM3	Model is high-resolution.	Model does not include sediment interaction and biological and physical-chemical processes.
INCA-Contaminant fate model x2	Model is high-resolution.	The model considers microplastics as pure and inert polymers.
	Model is accurate.	
Larval TRANSport	Model is high-resolution.	
Lagrangian model x2	Model is accurate.	
Telemac modelling system and BlueKenue.	Model is accurate	Low resolution

### Process-based modelling

Process-based models are comparable to hydrodynamic models since they also utilise hydrodynamic data but incorporate biological processes. There are relatively few studies that apply process-based modelling compared to purely hydrodynamic, which is due to the biological processes being difficult to obtain and considering extensive laboratory experiments. The models, like the hydrodynamic models, are high-resolution and precise, but the results depend on the ratio of scale and accuracy required. The main weakness of many process-based models is that they do not incorporate fragmentation of macroplastics, which is one of the major sources of microplastics in the aquatic environment. Biological inclusion may be one of the important drivers controlling the quantity and distribution of microplastics in the water column. This may deplete microplastics from the surface water and expedite microplastic burial, notably in summer seasons compared to winter. The distribution of microplastics varies seasonally with wind and currents and is dependent on the interaction of source locations, hydrodynamic conditions, degradation, settling and resuspension rates. The processes that most influence the accumulation of microplastics are the environmental conditions at the source locations, quantity, degradation rates, resuspension and settling rates. Sedimentation has a profound influence on microplastic transport and fate, followed by turbulent dispersion and beaching. The density, size and shape of the microplastics, influenced by biofouling thickness, also play a crucial role in the vertical transmission of particles. Particle size has a considerable influence on the retention of microplastics with retention being lowest for intermediate-sized particles. Low attachment efficiency results in a reduction of small particles. However, for intermediate and large-sized particles, it enhances retention. Biofilm also affects microplastic retention with particles ≥ 50 μm having no effect and particles ≤ 2 μm having their retention reduced from 60–50% to 50–40% (Table 3).

**Table 3:** Advantages and disadvantages of the reviewed process-based models

Process-based models	Advantages	Disadvantages
OxyDep, 2DBP and BioPlast models	Model considers microplastics as free particles, particles with biofouling, particles consumed and particles in detritus	Photo-, bio- and mechanical degradation is not considered
	Less computationally demanding	Stratification on the distribution of microplastics was not considered
	Model is high-resolution	
NanoDUFLOW model and R Studio software v0.98.976	Model is high-resolution	
	Model is accurate	
SLIM model	Highly versatile as it uses triangular elements	Model does not consider the effect of hills on islands and headlands
	Has fine-scale resolution at complex oceanography and coarse scale at open homogenous water movements	Computationally expensive
	Model is accurate at a local scale	
TrackMPD	Compatible with different formats of velocity inputs	Not compatible with unstructured grids
	Able to add complex and realistic physical processes	Does not consider fragmentation
	Model is high-resolution	
Modified version of the river multimedia box model	Model is high-resolution	
	Low computational effort	

### Statistical modelling

There were minimal studies that applied statistical modelling; however, the models were high-resolution and computationally efficient. The modelling framework is highly adaptable and can be employed in any aquatic setting. In terms of vulnerabilities, the models do have shortcomings, being the uncertainty of input values, simplified parameterization of beaching and sedimentation and vertical displacement, and so it is recommended that more realism be added to the analyses. However, the outcomes of statistical modelling appear to be fair. From the reviewed statistical modelling studies, the accumulation of plastic debris on the coastlines is nine times larger than at the bottom, which implies that the coastal area kinematics coupled with seasonality play a significant role in the fate of plastics. Statistically based modelling along with long-term integration of the Lagrangian transport equations has revealed that if beaching as a sink is solely considered, then the mean particle half-life is equal to 100 days. However, with sedimentation, the mean particle half-life is equal to 80 days (Table 4).

**Table 4:** Advantages and disadvantages of the reviewed statistical models

Statistical models	Advantages	Disadvantages
2D Markov chain model and the MEDSLIK-II model	Model is high-resolution	Limitation within-grid-cell correlation between entry and exit of particles
	Flexible	Degradation, sinking and ingestion by biota are not considered
	Computationally efficient	Simplified parameterisation of beaching and sedimentation of plastics
	Model allows forward-in-time and backwards-in-time simulation	

### Mass-balance modelling

The mass-balance models are precise and have high resolution, but they rely on spatial resolution and temporal scales; however, the simulated findings at the sub-catchment level are questionable due to the low resolution. The models are adaptable as they can be parameterised to represent rivers, dams or oceans. The models can be implemented in large regions, capturing high loads of microplastics entering the oceans and in environmental risk assessments, but there is uncertainty due to a lack of knowledge on emission rates and physicochemical properties. The models are not effective in local catchments as they exclude the river characteristics such as flow, river geometry and bathymetry. Furthermore, microplastic quantities differ across locations owing to varying socio-economic and technological features. From the reviewed mass-balance modelling studies, the fragmentation of macroplastics comprises one of the largest sources of microplastics from rivers, but this heavily depends on the assumed fragmentation rate. However, when results were compared to field measurement, they showed considerable variations, which is due to varying sampling methods and the uncertainty of mismanaged plastic waste values (Table 5).

**Table 5:** Advantages and disadvantages of the reviewed mass-balance models

Mass-balance models	Advantages	Disadvantages
Full Multi model	The modelling software is free	Overestimation of settling velocities and underestimation of the influence of particle shape
	The model can be readily parameterised to describe rivers, dams or oceans at different spatial and temporal scales	
	The model is accurate	
SimpleBox4nano	The model can calculate plastic concentration in air, soil, water and sediment	The modelling results are uncertain
Global NEWS model	Model is high-resolution	The modelling results are uncertain
Delft-3D WAQ	The model can be readily adapted to other watersheds	Model resolution is low at a sub-catchment level
(GREMiS) model	Model is high-resolution	The model does not provide detailed calculations of microplastic concentrations in individual catchments

### Machine learning modelling

Microplastic machine learning modelling has been given increasing attention in recent years, with the majority of the modelling based on the identification and classification of microplastics. The use of machine learning and image identification improves sample processing and accuracy of results, as highlighted in the reviewed articles, due to the replacement of the labour-intensive use of microscopic images. This has significantly reduced the time and error-related costs of conventional optical approaches, especially when multiple microplastic types are mixed in complex environmental media. All the reviewed machine learning studies proved to be accurate; however, the computational and data requirements were very high (Table 6).

**Table 6:** Advantages and disadvantages of the reviewed machine learning models

Machine learning models	Advantages	Disadvantages
ANN	Accurate	A lot of training is required
	High processing capability	Output is difficult to interpret
SVM	Models high dimensional numbers	A lot of training is required
	Increased sample size	
	Does not overfit	Lacking value sensitivity
Decision Tree	Accurate	Overfitting
	Simple and quick to train	
Random Forest	Accurate	Overfitting
	Simple and quick to train	
CNN	Accurate	Slow
	Quick	
	Can hold large amounts of data	Long training periods
KNN	Easy implementation	Does not work well with large datasets
	No training period	Sensitive to noise and missing data
U-Net	Accurate	Optimal depth is unknown
		Requires extensive architecture depth
PCA	No restrictions on parameters	Noise-sensitive
	Can remove noise	Information loss

### SWOT analysis

**Fig. 5:**
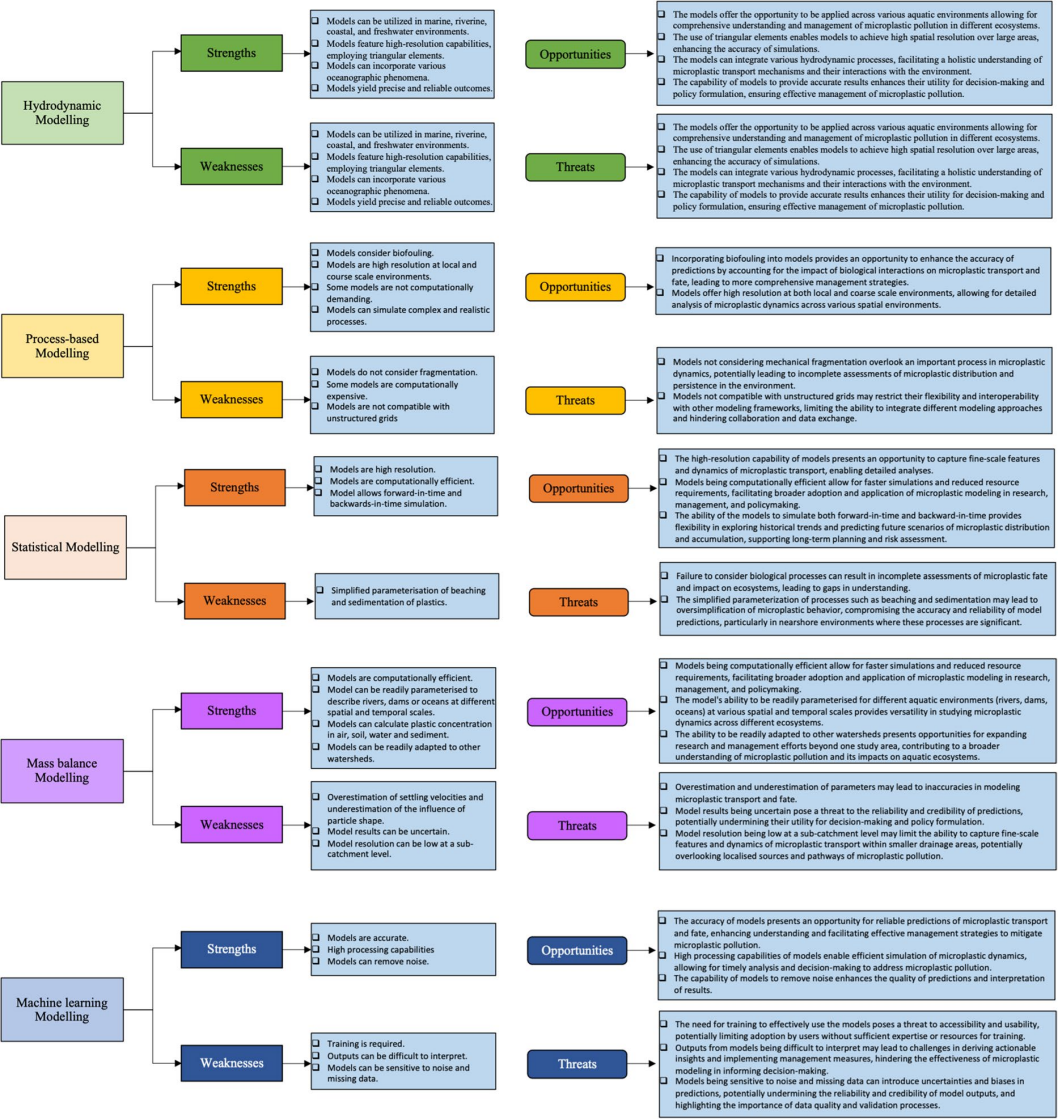
SWOT analysis of the analysed modelling frameworks (created on Microsoft PowerPoint)

This section presents strengths, weaknesses, opportunities and threats (SWOT) analysis of each modelling framework highlighting their opportunities and threats to aquatic microplastic transport and mitigation research. Figure 5 illustrates the overall strengths and weaknesses of each modelling type in simulating microplastic transport in the aquatic environment, of which their opportunities and threats were identified.

## Conclusions and future recommendations

It can be concluded that all modelling types resulted in relatively reliable findings, yet each modelling framework has its strengths and weaknesses in simulating microplastic transport and fate, as illustrated in Tables 1, 2, 3, 4, and 5.

Apart from the strengths and weaknesses of the modelling types, the reviewed models all had common issues such as inputs being unrealistically assumed, especially biological processes, as accurate inputs require extensive laboratory analysis; and there is a lack of sufficient field data for model calibration and validation, which therefore reduced model reliability. The paucity of data may be ascribed to in situ microplastic sampling and sample processing being expensive and there being a lack of standardised methodology for microplastic sampling. Where microplastic modelling studies included sampling on the river/seafloor, limited information was provided in terms of basic sedimentological information, viz. grain size, sediment type, depositional environment and water depth. This information should be included alongside microplastic information, viz. polymer type, size, shape and density as the information could go a long way in updating the global microplastic database, which will enable more efficient and accurate microplastic transport models to be developed. In terms of accessible published studies concentrating on modelling microplastics, relatively few apply process-based, statistical and mass-balance modelling, with the majority focusing on hydrodynamic processes. Also, the bulk of research generally concentrates on microplastic modelling in the oceans rather than rivers, dams and lakes. Microplastics may be effectively retained in these habitats, but within the oceans, there is a dearth of studies performed in the benthic areas. Where mass-balance models were applied, there was a lack of constraint on the sources and the transfer pathways of marine microplastics from shelf to ocean, which is critical in building credible global budgets.

In conclusion, this review identified several research recommendations to improve the reliability of microplastic transport and fate modelling being:

- In-depth research on the degradation rate of microplastics should be conducted to better determine realistic residence times, as deterioration to nanoscale size may affect the plastic’s transport properties.
- For more precise and reliable simulated results, a large amount of experimental and observational data is required.
- Because most modelling studies only use near-spherical shapes rather than fibres or sheets, the implications of the simulated microplastics’ physical shape must be investigated.
- Biofouling and aggregation must be modelled because they affect microplastic sedimentation, resulting in a better understanding of microplastic dispersal and ultimate fate. Additionally, turbulent conditions need to be considered.
- Future modelling research should incorporate the vertical mixing of microplastics in the water column.
- Additional research is required to characterise the transport and fate of microplastics in stormwater systems and treatment facilities.
- Due to a lack of defined procedures, harmonisation of sampling methodologies for surface and column waters is highly required, limiting the comparison of size categories, sample process, weight, and volume to a small number of published datasets.
- There should also be standardised units used to quantify the abundance of microplastics and studies should mention both mass and particle count data.
- The ecology of microbial life on microplastics should be investigated to comprehend the potential risk of pathogen dispersion with microplastic transport.
- To better characterise the origin of marine microplastics, simulations should include modelling of potential sources.
- More research regarding microplastic concentrations and characteristics needs to be conducted in freshwater environments.
- A database on microplastic structural features should be created, especially for the use of model training for machine learning modelling.
•More research regarding microplastic transport using machine learning algorithms needs to be conducted.

### Supplementary Information

Below is the link to the electronic supplementary material.

Supplementary file1 (DOCX 62 KB)

## Data Availability

Not applicable.
