# Supplementary material for: Applications of mathematical modelling for assessing microplastic transport and fate in water environments: a comparative review
Source: Environ Monit Assess. 2024 Jun 27;196(7):667. doi: 10.1007/s10661-024-12731-x (PMC11211188; doi:10.1007/s10661-024-12731-x)
Supplement: Supplementary file 1 — Supplementary file1 (DOCX 62 KB) [file 10661_2024_12731_MOESM1_ESM.docx]

**Table S1: Meta-analysis of articles employing the use of models to describe the transport of microplastics in the water environment.**

| **Author** | **Environment** | **Location** | **Aim/Objectives** | **Applied model** | **Model Type** | **Model input data** | **Main findings** |
| --- | --- | --- | --- | --- | --- | --- | --- |
| (Jalón-Rojas, Wang and Fredj 2019a) | Marine | Jervis Bay, Australia | The study aims to determine if 2-D modelling is sufficient for the accurate modelling of microplastic transport or if 3-D modelling is necessary. | The Particle Tracking and Analysis Toolbox for Matlab | Hydrodynamic | Flow velocity, temperature, salinity, and water level from the Princeton Ocean Model. | The 2-D simulation roughly reproduced the transport and accumulation patterns, but accurate results required a 3-D approach. |
| (Daily and Hoffman 2020) | Lake | Lake Erie | The study aims to model the impact of vertical movement of plastic particles. | Lagrangian transport model | Hydrodynamic | Current, diffusivity, wind, boundary conditions, population, and experimental sinking velocity data. | The results indicate that the accumulation pattern is driven by horizontal advection, however, when mixing is included, the plastics are distributed slightly more evenly through the water column. |
| (Atwood *et al.* 2019) | River | Po River,  Northern Italy | The study aims to model the coastal build-up of microplastic particles generated by the Po River. | Ichthyop lagrangian particle tracking model and a remote sensing model. | Hydrodynamic | Images of the Landsat 8, and the European Space Agency Sentinel-2 were used for the remote sensing model, and river discharge and wind speed were used in the hydrodynamic model. | The results indicate that the released particle quantity is semi-coupled to beaching rates, which are mouth dependent. Remote sensing better captured river mouth relative strength, and accumulation patterns were consistent with the hydrodynamic model. |
| (Collins and Hermes 2019) | Marine | South Africa | The study aims to model the accumulation of marine microplastics. | ICHTHYOP lagrangian particle tracking model and the Regional Ocean Modelling System. | Hydrodynamic | Boundary conditions, climate, temperature, salinity, and current velocity data. | Particles with a density lower than the density of seawater are either exported to the ocean or beached. |
| (Frere *et al.* 2017) | Marine | France – Bay of Brest | The study aims to model the influence of hydrodynamics on surface microplastic concentration and distribution. | Ichthyop v3.2 and the Model for Application at Regional Scale (MARS) 3D | Hydrodynamic | Seawater temperature and salinity, atmospheric pressure, wind speed, air temperature, relative humidity, sea elevation, river runoffs, tidal conditions and ocean currents. | The model results show the presence of a transitional convergence zone in the centre of the bay during flood tides. |
| (Hoffman and Hittinger 2017) | Lake | Laurentian Great Lakes | The study aims to estimate coastal plastic input and to model their transport use water current data. | Numerical model (4th order Runge-Kutta) and GLCFS model. | Hydrodynamic | Plastic debris data and the nowcast and forecast datasets. | The results derived surface microplastic mass estimates of 0.0211 metric tonnes in Lake Superior, 1.44 metric tonnes in Huron, and 4.41 metric tonnes in Erie. |
| (Enders *et al.* 2015) | Marine | European Coast to the North Atlantic Subtropical Gyre | The study aims to determine the quantity, size, and polymer type of microplastics and to model their vertical movement. | Numerical modelling | Hydrodynamic | Microplastic properties, temperature, salinity, advection, and diffusivity data. | The results indicate that once microplastics are fragmented to a certain size, distribution patterns can be observed horizontally and vertically. |
| (Bondelind *et al.* 2020) | River | Göta River, Sweden | The study aims to analyse the influence of size and density of tyre wear particles in road run-off. | MIKE 3 FM software | Hydrodynamic | Microplastic quantity, particle sizes, density and settling velocities. | Large quantities of microplastics were observed on the south side of the river owing to higher annual average daily traffic loads. The mixing processes in the river and the microplastic concentrations were influenced by the vertical water density gradient. |
| (Nizzetto *et al.* 2016) | River | Thames River - UK | The study aims to model microplastic transport across the pedosphere and hydrosphere. | INCA-Contaminant fate model. | Hydrodynamic | Data on the river geometry, sub-catchment boundaries, slopes, land use, and soil properties. | Microplastics of 0.2 mm are not retained, whereas larger microplastics with densities higher than water are retained in the sediment. However, high flow periods can cause resuspension. |
| (Mountford and Morales Maqueda 2019) | Marine | - | The study aims to model the dispersal of microplastics through the water column and the sea floor using ocean currents and turbulence. | Nucleus for European Modelling of the Ocean Version 3.6, configuration ORCA2‐LIM3. | Hydrodynamic | Plastic sampling data, viscosity, bathymetry, vertical mixing, and vertical viscosities. | The results reveal that there is a high accumulation of plastics within coastal areas and in the benthic areas. |
| (He *et al.* 2021) | River | Brisbane River, Australia | The study aims to model the transport processes of sedimental microplastics. | TUFLOW FV Particle Tracking Module | Hydrodynamic | Flow velocity, water levels, water temperature, salinity, wave and tidal data and sinking velocity data. | The study outcomes confirm that sedimental microplastics with low density have high mobility and high flow velocity in the bottom water layer of the water column enabling sedimental microplastic transport. |
| (Fossi *et al.* 2017) | Marine | Pelagos Sanctuary | The study aims at modelling the accumulation of hot spots of plastics. | Numerical circulation model and the Tyrreno- Regional Ocean Modelling System | Hydrodynamic | Topography, sea level anomaly, sea surface temperature and current data. | Areas with high microplastic density overlap with areas of high macroplastic density. The results also suggest that the primary source of microplastics is the fragmentation of macroplastics. |
| (Alosairi, Al-Salem and Al Ragum 2020) | Marine | North-western Arabian Gulf, Kuwait | The study aims to model the transport and fate of microplastics by analysing dominating wind conditions and reverse estuarine circulation. | The Delft3D-PART model was used with hydrodynamic data from the Delft3D-FLOW model. | Hydrodynamic | Water density, wind drag, horizontal viscosity, horizontal diffusivity, Secchi depth and Dalton number, taken from literature. | The findings indicate that the northerly winds influence the movement of microplastics in the open coastal zones, whereas in semi-enclosed areas they result in entrainment and beaching. |
| (Díez-Minguito *et al.* 2020) | Estuary | Spain | The study aims to model the distribution patterns of microplastics. | Idealized 2D-vertical model | Hydrodynamic | Meteorological and oceanographic conditions, sea level data, bathymetric data, and freshwater discharge records. | The results indicate that winds dominate the circulation in the outer part of the estuary, whereas near the head, the gravitational circulation takes over the control of the net ﬂow. |
| (Critchell *et al.* 2015) | Marine | Great Barrier Reef, Australia | The study aims to identify potential sites of plastic accumulation along the coastline. | SLIM model | Hydrodynamic | Current and wind data, seeding locations, river and ship input data, sea level and ocean surface topography data. | The orientation of beaches to the prevailing wind direction affected plastic accumulation. The wind drift coefﬁcient and the timing of plastic release minimally affected plastic transport in rivers but it greatly affected debris originating from ships. |
| (Zhang *et al.* 2022) | Marine | Yantai, China | The study aims to investigate an unexpected microplastic leakage event that occurred along the coastline. | Regional Ocean Modelling System (ROMS) and Larval TRANSport  Lagrangian model (LTRANS) | Hydrodynamic | Wind, boundary, and tidal data. | The modelling results show that the microplastics originate from the central and western parts of the Bohai Sea and are driven by northwest wind and wind-induced surface currents. |
| (Yu *et al.* 2018) | Marine | South-eastern United States | The objectives of the study are to quantify the microplastics and investigate the factors that inﬂuence their distribution. | Regional Ocean Modelling System and the Larval Transport  Lagrangian model | Hydrodynamic | Ocean hydrodynamic data, and microplastic quantity data. | The results indicate that the quantity of microplastics is generally related to the extent of nearby urbanisation. |
| (Genc, Vural and Balas 2020) | Marine | Fethiye Inner Bay, Turkey | The study aims to model the transport and accumulation of microplastics. | Transport submodule of HYDROTAM-3D | Hydrodynamic | Wind speed, wave climate, microplastic density and settling velocity, the density of the water, salinity, and water depth. | Microplastic accumulation is expected in the southwest coastal waters of the bay where coastal circulations are weak. |
| (Handyman *et al.* 2019) | Marine | North Indramayu, Java Sea | The study aims to investigate microplastic transport over seven months. | Mike 21 Flow Model FM of Mike 21 | Hydrodynamic | Bathymetry, current, wind, and microplastic data | The movement of microplastics has a circular reversing pattern caused by water currents. The estimated transport ranges of the microplastics were 0.9 – 5.4 km from the sampled location, seven months later. |
| (Eriksen *et al.* 2014) | Marine | The five sub-tropical gyres, coastal Australia, Bay of Bengal, and the Mediterranean Sea | The study aims to model the global distribution, count and weight of plastic waste in the world’s oceans. | HYCOM and Pol3DD models | Hydrodynamic | Net tow sample collection, ocean surface currents, wind stress, wind speed, heat flux and precipitation. | It was predicted that 5.25 trillion particles weighing 268 940 tonnes are in the oceans. |
| (Lebreton, Greer and Borrero 2012) | Marine | Global | The study presents a framework for describing the transport and accumulation of ﬂoating debris and the formation of oceanic accumulation zones. | Lagrangian particle  tacking model Pol3DD | Hydrodynamic | Sea surface currents, wind speed, temperature, precipitation and microplastic sampling data. | The simulation highlights the formation of ﬁve accumulation zones in the subtropical latitudes of the major ocean basins. |
| (Lebreton *et al.* 2017) | Marine | Global | The study aims to globally estimate river plastic inputs into the world’s oceans. | Global model | Hydrodynamic | Population density, mismanaged plastic waste and catchment runoff. | It was concluded that between 1.15 and 2.41 million tonnes of plastic waste enter the ocean from rivers annually. |
| (Whitehead *et al.* 2021) | River | River Thames-UK | The study aims to model the impacts of microplastics on river water quality and quantify the amount moving along the river. | Integrated catchments (INCA) microplastics model | Hydrodynamic | Microplastic data from efﬂuent discharges and sewage sludge disposal. | The results show that there is a significant amount of microplastics moving down the river with increasing deposition on the riverbed. |
| (Ding, Liu and Yang 2019) | Marine | Laizhou Bay, China | The study aims to investigate the movement trajectory of microplastic particles. | Lattice Boltzmann method and the Lagrangian particle-tracking method | Hydrodynamic | Wind, tidal and water depth data. | The results indicate that the particles drift within the scope of 4593 m × 8242 m near the releasing point and collisions have little effect on changing trajectories of particles. |
| (Ballent *et al.* 2013) | Marine | Nazaré Canyon, Portugal | The study aims to predict microplastic transport pathways and likely accumulation hotspots. | MOHID Water Model System | Hydrodynamic | Experimental shear-stress, settling velocity and resuspension data. | The model results indicate that microplastic transport was greater during autumn and winter due to occasional storm activity and internal wave action. |
| (Cook *et al.* 2020) | River | - | The study aims to track the movement of both fluorescent dye and florescent stained microplastics in laboratory flumes with standard fibreoptic fluorometers using fluorometric principles. | Experimental | Hydrodynamic | Neutrally buoyant microplastics and Rhodamine dye. | Neutrally buoyant microplastics behave like rhodamine and display fundamental dispersion theory. This suggests Rhodamine can be released into the natural environment to replicate microplastic transport in the water column. |
| (Babajamaaty, Mohammadian and Pilechi 2022) | River | Fraser River, Georgia | The study aims to model the transport of microplastics in a highly urbanised and industrialised area. | Telemac modelling system and BlueKenue. | Hydrodynamic | Bathymetry data, water level, flow velocity, salinity | The results indicated that the hydrodynamic mesh solution in both horizontal and vertical directions, the discharge rate upstream, time step size, boundary treatment method and quality of the bathymetry data were the main factors which influenced the behaviour of the particles. |
| (Li *et al.* 2018) | Marine | Bohai Sea,  China | The study aims to investigate the transport processes of plastic particles through numerical experiments. | SLIM model | Process-based | Sea level, mean current, and wind data. | Microplastic distribution varies seasonally and depends on the source locations, hydrodynamic conditions, degradation, settling and resuspension rates |
| (Critchell and Lambrechts 2016) | Marine | Whitsunday Islands, Australia | The study aims to model the importance of the physical processes governing plastic accumulation. | SLIM model | Process-based | Resuspension rate, degradation rate, wind shadow, length of wind shadow, wind drift coefficient, settling rate, source location and diffusivity. | The results indicate that the processes that significantly influence plastic accumulation on beaches are the source locations, quantity, degradation, and resuspension. |
| (Sani-Kast *et al.* 2015) | River | Rhone River-France | The study aims to investigate the influence of various parameters on the fate of engineered nanoplastics. | A modified version of the river multimedia box model | Process-based | Water composition data, river morphology and flow velocity. | The results indicate that the fate of nanoparticles can be described by relatively small concentrations and is significantly dependent on conditions near their seeding source. |
| (Jalón-Rojas, Wang and Fredj 2019b) | Marine | Jervis Bay-Australia | The study aims to develop a TrackMPD modelling framework that simulates the main physical processes affecting microplastic behaviours. | TrackMPD | Process-based | Advection, dispersion, windage, sinking, settling, beaching and re-floating. | The microplastic dynamical properties that impact their sinking, in particular, plastic density and biofilm thickness and density, have the biggest effect on the microplastic transport, followed by turbulent dispersion and washing-off. |
| (Berezina *et al.* 2021) | Marine | Oslo Fjord, Norway | The study aims at determined why denser plastics can be found in the water column while lighter ones are found in sediments. | OxyDep, 2DBP and BioPlast models | Process-based | Microplastic input data, boundary conditions and wind data. | Biological inclusion was found to be one of the important drivers controlling the quantity and transport of microplastics. The inclusion depleted microplastics from the surface and accelerated burying in summer compared to the winter. |
| (Besseling *et al.* 2017) | River | Dommel River, Netherlands | The study aims to analyse the fate and retention of plastic in the river. | NanoDUFLOW model and R Studio software v0.98.976 | Process-based | Experimental attachment efficiency, sedimentation, aggregation, degradation, resuspension, and burial data. | The data shows that particle size significantly influences the simulated retention and accumulation hot spots in the sediment, with retention being lowest for intermediate-sized particles (5 μm). |
| (Coppini *et al.* 2018) | Marine | Adriatic Sea | The study aims to model the contribution of microplastic beaching, sedimentation, fragmentation, and uptake of biota. | 2D Markov chain model and the MEDSLIK-II model | Statistical | Ocean current, wind, environmental monitoring service data and microplastic inputs. | The results indicate that it will take 90 days to populate the Mediterranean basin with virtual microplastics and if beaching as a sink is only considered then the mean particle half-life will be 100 days, however, with sedimentation the mean particle half-life will be 80 days. |
| (Liubartseva *et al.* 2016) | Marine | Adriatic Sea | The study aims to simulate the plastic concentrations at the sea surface and ﬂuxes onto the coastline. | Markov chain model and MEDSLIK-I lagrangian model | Statistical | Terrestrial and maritime plastic inputs, ocean currents and wind data. | The coastline receives a plastic ﬂux of approximately 70 kg/day. |
| (Liubartseva *et al.* 2018) | Marine | Mediterranean Sea | The study aims to model the distribution of plastic debris at the sea surface, on the coastlines and at the  sea bottom. | 2D lagrangian Markov chain model | Statistical | Ocean current and wave data. | Plastic accumulation was identified on the coastlines and the sea bottom, and highly polluted zones in the vicinity of sources were identiﬁed. |
| (Maximenko, Hafner and Niiler 2012) | Marine | - | The study aims to use the global set of historical trajectories of drifting buoys to measure surface currents. | Stochastic model | Statistical | Drifters released | The study reveals five main sites of drifter aggregation, located in the subtropics and maintained by converging Ekman current. |
| (Siegfried *et al.* 2017) | River | European Rivers | This study aims to estimate microplastic fluxes from land to sea. | Global NEWS (Nutrient Export from WaterSheds) model. | Mass-balance | Microplastic estimates, land use, agricultural and socio-economic parameters. | The study did not provide a validated model. However, the results indicate that synthetic polymers from tyres and road wear comprise the largest source of microplastic pollution. |
| (Mai *et al.* 2019) | River | Pearl River Delta, China | The study aims to conduct sampling to provide field-measured data for validating modelling results. | Mass-balance model | Mass-balance | Microplastic field data | The annual riverine input of microplastics was estimated at 2400–3800 tons of plastic debris. These values were substantially below the mismanaged plastic waste-based model estimates (91,000–170,000 tons). The large difference between measured and modelled results may have derived from the large uncertainty in the mismanaged plastic waste values assigned to the world’s countries/regions. |
| (van Wijnen, Ragas and Kroeze 2019) | River | Global | This study aims to contribute to literature on the export of microplastics from land to sea. | Global Riverine Export of  Microplastics into Seas (GREMiS) model | Mass-balance | Microplastic inputs, land use data and river hydrology data. | The results indicate that fragmentation of macroplastics is the primary source of microplastics, but depends on the fragmentation rate. |
| (Unice *et al.* 2019) | River | Seine River-France | The study aims to develop a mass balance model to assess the fate of tyre and road wear particles. | Delft-3D WAQ | Mass-balance | Tyre wear and generation rate, particle density, particle diameter, biofilm thickness and density, | The modelled pseudo steady state sediment concentrations were consistent with measurements from the Seine watershed supporting the plausibility of the predicted trapping efﬁciency of approximately 90%. |
| (Koelmans *et al.* 2017) | Marine | - | The study aims to model the global ocean plastic inventory. | Mass-balance equations | Mass-balance | Plastic inputs, fragmentation rates and settling rate. | The results indicate that 99.8% of the plastics that had entered the ocean since 1950 had settled below the ocean surface level by 2016. |
| (Domercq, Praetorius and MacLeod 2022) | River | - | The study aims to model the fate of plastic particles by describing particle size. | Full Multi model | Mass-balance model | Particle size, fragmentation, biofouling, attachment efficiency, degradation, and river discharge data. | The model predicts a maximum abundance of floating plastics at 1 μm diameter. |
| (Meesters *et al.* 2014) | Air, soil & water | Switzerland | The study aims to model the behaviour of engineered nanoparticles and to evaluate their potential for use in environmental risk assessment. | SimpleBox4nano (SB4N) | Mass-balance | Data on attachment rates, collision frequency, aggregation, and attachment frequency. | The results indicate that the model can be implemented in environmental risk assessment frameworks. |
| (Guo and Wang 2021) | Marine, lakes and rivers | Global | The study aims to predict the sorption capacity of heavy metal ions onto microplastics in global aquatic environments | Back-propagation ANN model and the NN toolbox of Matlab R2017a | Machine learning | Sorption data of heavy metal ions (Cd, Pb, Cr, Cu, Zn) onto microplastics and salinity. | The model was able to predict the sorption capacity of heavy metal ions with high R values (0.926-0.994). the predicted sorption capacity in rivers and lakes was higher than in the ocean. |
| (Yurtsever and Yurtsever 2019) | - | Sakarya, Turkey | The study aims to achieve automatic classification of microplastics based on microscopic images. | Convolutional neural network (CNN) and GoogleNet architecture | Machine learning | Microscope images of microplastics from water and wastewater samples. | The results indicated that CNN achieved a classification performance of 89% for microplastics in wastewater. |
| (Lee *et al.* 2022) | River | Langat River, Malaysia | The study aims at using machine learning techniques to automatically segment and count microplastics in a given image. | U-Net convolutional neural network | Machine learning | Images of microplastics sieved through a 53 μm mesh | The results indicate that U-Net can achieve human-level performance in counting microplastics in cluttered images. |
| (Lorenzo-Navarro *et al.* 2021) | Marine | Tenerife, Spain | The study aims to automatically count and classify microplastics from pictures. | U-Net neural network and VGG16 neural network. | Machine learning | Images of microplastics taken from digital cameras and a mobile phone. | The results indicated that the proposed models had a 98.11% accuracy in the classification of microplastics. |
| (Bianco *et al.* 2020) | Marine | - | The study aims at replacing unaided microscope observation of pretreated water samples with an automated prescreening tool. | Digital holography and support vector machine (SVM) | Machine learning | Microplastic data | The results indicate that holography and machine learning improves microplastic recognition in heterogeneous samples and achieved 99% accuracy. |
| (Bifano *et al.* 2022) | - | - | The study aims at determining the presence and type of microplastics in water samples. | Electrical impedance spectroscopy and SVM. | Machine learning | Microplastics | The results indicate that microplastics can be distinguished securely and the concentrations can be estimated quantitatively. |
| (de Medeiros Back *et al.* 2022) | Marine | Mediterranean Sea | The study aims to demonstrate the performance of different machine-learning classification algorithms to classify ocean microplastics. | Support Vector Machine Classifier | Machine learning | Microplastic data from the Attenuated Total Reflection FTIR spectroscopy. | Support Vector Machine Classifier provided a good relationship between simplicity and performance, for fast automatic characterisation of microplastics. |
| (Meyers *et al.* 2022) | Marine | Global | The study aims at developing a method to detect and identify microplastics. | Decision tree models (Plastic Detection Model And Polymer Identification Model) | Machine learning | Photos of Nile red-fluorescently stained microplastics | The models predicted with high accuracy the plastic or natural origin of particles (95.8%), and the polymer types of the microplastics (88.1%). |
| (Lorenzo-Navarro *et al.* 2018) | - | - | The study aims to automatically count and classify microplastic particles using Computer Vision and machine learning techniques. | K Nearest-Neighbor, C4.5, Random Forest, Adaptive Boosting and Support Vector Machine. | Machine learning | Images of microplastics. | The results indicated that by making use of colour-based and shape-based features along with a Random Forest classifier, an accuracy of 96.6% was achieved in recognising microplastics. |
| (Jiang *et al.* 2022) | Estuary | China | The study aims to predict the relative abundance of Vibrio spp on microplastics using machine learning models | Support vector regression (SVR), Deep Neural Network, RandomForest, ElasticNet, and XGBoost models | Machine learning | Microplastic samples | The results indicated that the DNN model and RandomForest algorithm achieved the best predictive performance. |
| (Lei *et al.* 2022) | - | - | The study aims at demonstrating near-quantitative classification accuracy of Raman spectra using three different machine learning algorithms. | Random-forest, K-nearest neighbors and multi-layer perception algorithms in scikit-learn python package. | Machine learning | 186 high-resolution Raman spectra | The results indicate that Raman spectra and machine learning techniques are capable of producing classification models that yield>95% classification accuracy. |
| (Kedzierski *et al.* 2019) | Marine | Mediterranean Sea | The study aims to determine if the K-nearest neighbors classification method can be used for automated identification of FTIR spectra of microplastics. | K-nearest neighbors classification | Machine learning | Spectra of microplastics | The results indicate that the machine learning process is efficient in identifying spectra of polymers such as polyethylene. |
| (Michel *et al.* 2020) | Marine | Global | The study aims at identifying domestic and ocean microplastics using a combination of spectroscopic techniques and machine learning classifiers. | KNN, LDA, SVM and PCA. | Machine learning | Microplastic samples | The classification of microplastics from the ocean had lower success rates than domestic microplastics, arising from alterations due to environmental weathering processes with success rates of 99, 81, 76, and 66% for ATR−FTIR, and NIR reflectance spectroscopy. |
| (da Silva *et al.* 2020) | - | Denmark | The study aims to develop an automated analytical method for the characterisation of microplastics using micro-Fourier transform infrared hyperspectral imaging and machine learning. | Partial least squares discriminant analysis (PLS-DA) and soft independent modelling of class analogy (SIMCA). | Machine learning | Microplastics data | PLS-DA presented a better analytical performance in comparison with SIMCA models with higher sensitivity, sensibility, and lower misclassification error. |
| (Yan *et al.* 2022) | Marine | Mediterranean coast | The study aims to evaluate the performance of 7 machine learning-based approaches in identifying and characterising microplastics using a standard FTIR spectral dataset. | Principal Component Analysis (PCA), ANN, Random Forests (RF), The K-Nearest Neighbors (KNN), Linear Discriminant Analysis, SVM, Partial Least Squares Discriminant Analysis (PLSDA) and Soft Independent Modelling of Class Analogies (SIMCA) | Machine learning | Microplastic spectral datasets | The results show that the imbalanced sample size in the dataset and microplastic fouling effects have significant impacts on the performance of the machine learning algorithms for identifying microplastic types. |
| (Chaczko *et al.* 2019) | - | - | The study aims at locating and classifying microplastics from hyperspectral images using machine learning. | Neaural network model using Python language and Tensorflow for building Neaural Networks. | Machine learning | Hyperspectral images of microplastics “SpecTex”. | The results of the model resulted in an accuracy of 95%. |
| (Luo *et al.* 2022) | Marine, lakes, rivers and potable water | - | The study aims to use machine learning combined with Raman spectroscopy to achieve rapid analysis of microplastics in different water environments. | Raman spectroscopy and Sparse-Autoencoder built on PyCharm. | Machine learning | Microplastic particles | The results indicate that microplastic types were identified with a 99.1% success rate. |

**Table S2: Model input parameter checklist.**

| **Articles** | **Model** | **Shear stress/drag coefficient** | **Salinity** | **Biofouling** | **Aggregation** | **Degradation/fragmentation/mechanical breakdown** | **Advection** | **Diffusion** | **Windage** | **Sedimentation-Resuspension** | **Beaching** |
| --- | --- | --- | --- | --- | --- | --- | --- | --- | --- | --- | --- |
| (Alosairi, Al-Salem and Al Ragum 2020) | The Delft3D-PART | ✓ | ✓ |  |  |  | ✓ | ✓ | ✓ |  | ✓ |
| (Atwood *et al.* 2019) | Ichthyop |  | ✓ |  |  |  | ✓ | ✓ | ✓ |  | ✓ |
| (Babajamaaty, Mohammadian and Pilechi 2022) | Telemac modelling system and BlueKenue. | ✓ | ✓ |  |  |  | ✓ | ✓ |  |  |  |
| (Ballent *et al.* 2013) | MOHID Water Model System | ✓ | ✓ | ✓ |  |  | ✓ | ✓ | ✓ | ✓ |  |
| (Berezina *et al.* 2021) | OxyDep, 2DBP and BioPlast |  | ✓ | ✓ |  |  | ✓ | ✓ | ✓ |  |  |
| (Besseling *et al.* 2017) | NanoDUFLOW | ✓ |  | ✓ | ✓ | ✓ | ✓ | ✓ | ✓ | ✓ |  |
| (Bondelind *et al.* 2020) | MIKE 3 FM |  | ✓ |  |  |  | ✓ | ✓ | ✓ |  |  |
| (Collins and Hermes 2019) | Ichthyop |  | ✓ |  |  |  | ✓ | ✓ | ✓ | ✓ | ✓ |
| (Cook *et al.* 2020) | Experimental | ✓ |  |  |  |  | ✓ | ✓ |  |  |  |
| (Coppini *et al.* 2018) | 2D Markov chain and the MEDSLIK-II model |  |  |  |  |  | ✓ | ✓ | ✓ | ✓ | ✓ |
| (Critchell *et al.* 2015) | SLIM | ✓ |  |  |  |  | ✓ | ✓ | ✓ |  | ✓ |
| (Critchell and Lambrechts 2016) | SLIM |  |  |  |  | ✓ | ✓ | ✓ | ✓ | ✓ | ✓ |
| (Daily and Hoffman 2020) | Lagrangian transport model |  |  |  |  |  | ✓ | ✓ | ✓ | ✓ | ✓ |
| (Díez-Minguito *et al.* 2020) | Idealized 2D-vertical model | ✓ | ✓ |  |  |  | ✓ | ✓ | ✓ | ✓ | ✓ |
| (Ding, Liu and Yang 2019) | Lattice Boltzmann method and the Lagrangian particle-tracking method | ✓ |  |  |  |  | ✓ |  | ✓ |  |  |
| (Domercq, Praetorius and MacLeod 2022) | Full Multi model | ✓ |  | ✓ | ✓ | ✓ | ✓ | ✓ | ✓ | ✓ |  |
| (Enders *et al.* 2015) | Numerical modelling | ✓ | ✓ |  |  | ✓ | ✓ | ✓ | ✓ | ✓ |  |
| (Eriksen *et al.* 2014) | HYCOM and Pol3DD models |  |  |  |  | ✓ | ✓ | ✓ | ✓ |  |  |
| (Fossi *et al.* 2017) | Numerical circulation model and the Tyrreno- Regional Ocean Modelling System |  |  |  |  | ✓ | ✓ | ✓ | ✓ |  |  |
| (Frere *et al.* 2017) | Ichthyop v3.2 and the Model for Application at Regional Scale (MARS) 3D |  | ✓ |  | ✓ |  | ✓ |  | ✓ | ✓ | ✓ |
| (Genc, Vural and Balas 2020) | Transport submodule of HYDROTAM-3D | ✓ | ✓ |  |  |  | ✓ | ✓ | ✓ | ✓ |  |
| (Handyman *et al.* 2019) | Mike 21 Flow Model FM of Mike 21 |  |  |  |  |  | ✓ |  | ✓ |  |  |
| (He *et al.* 2021) | TUFLOW FV Particle Tracking Module | ✓ | ✓ |  |  |  | ✓ | ✓ |  | ✓ |  |
| (Hoffman and Hittinger 2017) | Numerical model (4th order Runge-Kutta) and GLCFS model. |  |  |  |  |  | ✓ |  | ✓ |  |  |
| (Jalón-Rojas, Wang and Fredj 2019a) | The Particle Tracking and Analysis Toolbox | ✓ | ✓ | ✓ |  | ✓ | ✓ | ✓ | ✓ | ✓ | ✓ |
| (Jalón-Rojas, Wang and Fredj 2019b) | TrackMPD | ✓ | ✓ |  |  |  | ✓ | ✓ | ✓ | ✓ | ✓ |
| (Koelmans *et al.* 2017) | Mass-balance equations |  |  | ✓ | ✓ | ✓ |  |  |  | ✓ |  |
| (Lebreton *et al.* 2017) | Global model |  |  |  |  |  |  |  |  |  |  |
| (Lebreton, Greer and Borrero 2012) | Lagrangian particle  tacking model Pol3DD |  |  |  |  |  | ✓ | ✓ | ✓ |  |  |
| (Li *et al.* 2018) | SLIM model |  |  |  |  | ✓ | ✓ | ✓ | ✓ | ✓ | ✓ |
| (Liubartseva *et al.* 2016) | Markov chain model and MEDSLIK-I lagrangian model |  |  |  |  |  | ✓ | ✓ | ✓ |  |  |
| (Liubartseva *et al.* 2018) | 2D lagrangian Markov chain model |  | ✓ | ✓ | ✓ | ✓ | ✓ | ✓ | ✓ | ✓ | ✓ |
| (Mai *et al.* 2019) | Mass-balance model |  |  |  |  |  | ✓ |  |  | ✓ |  |
| (Maximenko, Hafner and Niiler 2012) | Stochastic model |  |  |  | ✓ |  | ✓ | ✓ | ✓ |  |  |
| (Meesters *et al.* 2014) | SimpleBox4nano (SB4N) | ✓ |  | ✓ | ✓ | ✓ | ✓ | ✓ |  | ✓ |  |
| (Mountford and Morales Maqueda 2019) | Nucleus for European Modelling of the Ocean Version 3.6, configuration ORCA2‐LIM3. |  | ✓ |  |  |  | ✓ | ✓ |  | ✓ |  |
| (Nizzetto *et al.* 2016) | INCA-Contaminant fate model. | ✓ |  |  |  | ✓ | ✓ |  |  | ✓ |  |
| (Sani-Kast *et al.* 2015) | A modified version of the river multimedia box model |  |  |  | ✓ |  | ✓ |  |  | ✓ |  |
| (Siegfried *et al.* 2017) | Global NEWS |  |  |  |  |  | ✓ |  | ✓ |  |  |
| (Unice *et al.* 2019) | Delft-3D WAQ | ✓ |  | ✓ | ✓ | ✓ | ✓ | ✓ |  | ✓ |  |
| (van Wijnen, Ragas and Kroeze 2019) | Global Riverine Export of  Microplastics into Seas (GREMiS) model |  |  | ✓ | ✓ | ✓ | ✓ |  | ✓ | ✓ |  |
| (Whitehead *et al.* 2021) | Integrated catchments (INCA) microplastics model |  |  |  |  |  | ✓ |  |  | ✓ |  |
| (Yu *et al.* 2018) | Regional Ocean Modelling System and the Larval Transport  Lagrangian model |  | ✓ |  |  | ✓ | ✓ |  |  |  |  |
| (Zhang *et al.* 2022) | Regional Ocean Modelling System and the Larval Transport  Lagrangian model | ✓ | ✓ |  |  |  | ✓ | ✓ | ✓ | ✓ |  |

**References:**

Alosairi, Y., Al-Salem, S. and Al Ragum, A. 2020. Three-dimensional numerical modelling of transport, fate and distribution of microplastics in the northwestern Arabian/Persian Gulf. *Marine pollution bulletin*, 161: 111723.

Atwood, E. C., Falcieri, F. M., Piehl, S., Bochow, M., Matthies, M., Franke, J., Carniel, S., Sclavo, M., Laforsch, C. and Siegert, F. 2019. Coastal accumulation of microplastic particles emitted from the Po River, Northern Italy: comparing remote sensing and hydrodynamic modelling with in situ sample collections. *Marine pollution bulletin*, 138: 561-574.

Babajamaaty, G., Mohammadian, A. and Pilechi, A., 2021, May. Numerical Modeling of Microplastics Fate and Transport in a Stretch of the Fraser River. In Canadian Society of Civil Engineering Annual Conference (pp. 99-107). Singapore: Springer Nature Singapore.

Ballent, A., Pando, S., Purser, A., Juliano, M. and Thomsen, L. 2013. Modelled transport of benthic marine microplastic pollution in the Nazaré Canyon. *Biogeosciences*, 10 (12): 7957-7970.

Berezina, A., Yakushev, E., Savchuk, O., Vogelsang, C. and Staalstrom, A. 2021. Modelling the influence from biota and organic matter on the transport dynamics of microplastics in the water column and bottom sediments in the oslo fjord. *Water*, 13 (19): 2690.

Besseling, E., Quik, J. T., Sun, M. and Koelmans, A. A. 2017. Fate of nano-and microplastic in freshwater systems: A modeling study. *Environmental Pollution*, 220: 540-548.

Bianco, V., Memmolo, P., Carcagnì, P., Merola, F., Paturzo, M., Distante, C. and Ferraro, P. 2020. Microplastic identification via holographic imaging and machine learning. *Advanced Intelligent Systems*, 2 (2): 1900153.

Bifano, L., Meiler, V., Peter, R. and Fischerauer, G. 2022. Detection of microplastics in water using electrical impedance spectroscopy and support vector machines. In: Proceedings of *Sensors and Measuring Systems; 21th ITG/GMA-Symposium*. VDE, 1-4.

Bondelind, M., Sokolova, E., Nguyen, A., Karlsson, D., Karlsson, A. and Björklund, K. 2020. Hydrodynamic modelling of traffic-related microplastics discharged with stormwater into the Göta River in Sweden. *Environmental Science and Pollution Research*, 27 (19): 24218-24230.

Chaczko, Z., Wajs-Chaczko, P., Tien, D. and Haidar, Y. 2019. Detection of microplastics using machine learning. In: Proceedings of *2019 International Conference on Machine Learning and Cybernetics (ICMLC)*. IEEE, 1-8.

Collins, C. and Hermes, J. 2019. Modelling the accumulation and transport of floating marine micro-plastics around South Africa. *Marine pollution bulletin*, 139: 46-58.

Cook, S., Chan, H.-L., Abolfathi, S., Bending, G. D., Schäfer, H. and Pearson, J. M. 2020. Longitudinal dispersion of microplastics in aquatic flows using fluorometric techniques. *Water research*, 170: 115337.

Coppini, G., Liubartseva, S., Lecci, R., Cretì, S., Verri, G., Clementi, E. and Pinardi, N. 2018. Toward 3D modeling the plastic marine debris in the Mediterranean. In: Proceedings of *Proceedings of the International Conference on Microplastic Pollution in the Mediterranean Sea*. Springer, 37-45.

Critchell, K., Grech, A., Schlaefer, J., Andutta, F., Lambrechts, J., Wolanski, E. and Hamann, M. 2015. Modelling the fate of marine debris along a complex shoreline: Lessons from the Great Barrier Reef. *Estuarine, Coastal and Shelf Science*, 167: 414-426.

Critchell, K. and Lambrechts, J. 2016. Modelling accumulation of marine plastics in the coastal zone; what are the dominant physical processes? *Estuarine, Coastal and Shelf Science*, 171: 111-122.

da Silva, V. H., Murphy, F., Amigo, J. M., Stedmon, C. and Strand, J. 2020. Classification and quantification of microplastics (< 100 μm) using a focal plane array–Fourier transform infrared imaging system and machine learning. *Analytical Chemistry*, 92 (20): 13724-13733.

Daily, J. and Hoffman, M. J. 2020. Modeling the three-dimensional transport and distribution of multiple microplastic polymer types in Lake Erie. *Marine pollution bulletin*, 154: 111024.

de Medeiros Back, H., Junior, E. C. V., Alarcon, O. E. and Pottmaier, D. 2022. Training and evaluating machine learning algorithms for ocean microplastics classification through vibrational spectroscopy. *Chemosphere*, 287: 131903.

Díez-Minguito, M., Bermúdez, M., Gago, J., Carretero, O. and Viñas, L. 2020. Observations and idealized modelling of microplastic transport in estuaries: the exemplary case of an upwelling system (Ría de Vigo, NW Spain). *Marine Chemistry*, 222: 103780.

Ding, Y., Liu, H. and Yang, W. 2019. Numerical Prediction of the Short-Term Trajectory of Microplastic Particles in Laizhou Bay. *Water*, 11 (11): 2251.

Domercq, P., Praetorius, A. and MacLeod, M. 2022. The Full Multi: An open-source framework for modelling the transport and fate of nano-and microplastics in aquatic systems. *Environmental Modelling & Software*, 148: 105291.

Enders, K., Lenz, R., Stedmon, C. A. and Nielsen, T. G. 2015. Abundance, size and polymer composition of marine microplastics≥ 10 μm in the Atlantic Ocean and their modelled vertical distribution. *Marine pollution bulletin*, 100 (1): 70-81.

Eriksen, M., Lebreton, L. C., Carson, H. S., Thiel, M., Moore, C. J., Borerro, J. C., Galgani, F., Ryan, P. G. and Reisser, J. 2014. Plastic pollution in the world's oceans: more than 5 trillion plastic pieces weighing over 250,000 tons afloat at sea. *PloS one*, 9 (12): e111913.

Fossi, M. C., Romeo, T., Baini, M., Panti, C., Marsili, L., Campani, T., Canese, S., Galgani, F., Druon, J.-N. and Airoldi, S. 2017. Plastic debris occurrence, convergence areas and fin whales feeding ground in the Mediterranean marine protected area Pelagos sanctuary: a modeling approach. *Frontiers in Marine Science*: 167.

Frere, L., Paul-Pont, I., Rinnert, E., Petton, S., Jaffré, J., Bihannic, I., Soudant, P., Lambert, C. and Huvet, A. 2017. Influence of environmental and anthropogenic factors on the composition, concentration and spatial distribution of microplastics: a case study of the Bay of Brest (Brittany, France). *Environmental Pollution*, 225: 211-222.

Genc, A. N., Vural, N. and Balas, L. 2020. Modeling transport of microplastics in enclosed coastal waters: A case study in the Fethiye Inner Bay. *Marine pollution bulletin*, 150: 110747.

Guo, X. and Wang, J. 2021. Projecting the sorption capacity of heavy metal ions onto microplastics in global aquatic environments using artificial neural networks. *Journal of hazardous materials*, 402: 123709.

Handyman, D. I. W., Purba, N. P., Pranowo, W. S., Harahap, S. A., Dante, I. F. and Yuliadi, L. P. S. 2019. Microplastics Patch Based on Hydrodynamic Modeling in The North Indramayu, Java Sea. *Polish Journal of Environmental Studies*, 28 (1)

He, B., Smith, M., Egodawatta, P., Ayoko, G. A., Rintoul, L. and Goonetilleke, A. 2021. Dispersal and transport of microplastics in river sediments. *Environmental Pollution*, 279: 116884.

Hoffman, M. J. and Hittinger, E. 2017. Inventory and transport of plastic debris in the Laurentian Great Lakes. *Marine pollution bulletin*, 115 (1-2): 273-281.

Jalón-Rojas, I., Wang, X.-H. and Fredj, E. 2019a. On the importance of a three-dimensional approach for modelling the transport of neustic microplastics. *Ocean Science*, 15 (3): 717-724.

Jalón-Rojas, I., Wang, X. H. and Fredj, E. 2019b. A 3D numerical model to track marine plastic debris (TrackMPD): sensitivity of microplastic trajectories and fates to particle dynamical properties and physical processes. *Marine pollution bulletin*, 141: 256-272.

Jiang, J., Zhou, H., Zhang, T., Yao, C., Du, D., Zhao, L., Cai, W., Che, L., Cao, Z. and Wu, X. E. 2022. Machine learning to predict dynamic changes of pathogenic Vibrio spp. abundance on microplastics in marine environment. *Environmental Pollution*, 305: 119257.

Kedzierski, M., Falcou-Préfol, M., Kerros, M. E., Henry, M., Pedrotti, M. L. and Bruzaud, S. 2019. A machine learning algorithm for high throughput identification of FTIR spectra: Application on microplastics collected in the Mediterranean Sea. *Chemosphere*, 234: 242-251.

Koelmans, A. A., Kooi, M., Law, K. L. and Van Sebille, E. 2017. All is not lost: deriving a top-down mass budget of plastic at sea. *Environmental Research Letters*, 12 (11): 114028.

Lebreton, L., Van Der Zwet, J., Damsteeg, J.-W., Slat, B., Andrady, A. and Reisser, J. 2017. River plastic emissions to the world’s oceans. *Nature communications*, 8 (1): 1-10.

Lebreton, L.-M., Greer, S. and Borrero, J. C. 2012. Numerical modelling of floating debris in the world’s oceans. *Marine pollution bulletin*, 64 (3): 653-661.

Lee, K. S., Chen, H. L., Ng, Y. S., Maul, T., Gibbins, C., Ting, K.-N., Amer, M. and Camara, M. 2022. U-Net skip-connection architectures for the automated counting of microplastics. *Neural Computing and Applications*, 34 (9): 7283-7297.

Lei, B., Bissonnette, J. R., Hogan, Ú. E., Bec, A. E., Feng, X. and Smith, R. D. 2022. Customizable Machine-Learning Models for Rapid Microplastic Identification Using Raman Microscopy. *Analytical Chemistry*, 94 (49): 17011-17019.

Li, Y., Wolanski, E., Dai, Z., Lambrechts, J., Tang, C. and Zhang, H. 2018. Trapping of plastics in semi-enclosed seas: Insights from the Bohai Sea, China. *Marine pollution bulletin*, 137: 509-517.

Liubartseva, S., Coppini, G., Lecci, R. and Clementi, E. 2018. Tracking plastics in the Mediterranean: 2D Lagrangian model. *Marine pollution bulletin*, 129 (1): 151-162.

Liubartseva, S., Coppini, G., Lecci, R. and Creti, S. 2016. Regional approach to modeling the transport of floating plastic debris in the Adriatic Sea. *Marine pollution bulletin*, 103 (1-2): 115-127.

Lorenzo-Navarro, J., Castrillón-Santana, M., Gómez, M., Herrera, A. and Marín-Reyes, P. A. 2018. Automatic counting and classification of microplastic particles. In: Proceedings of *ICPRAM 2018-Proceedings of the 7th International Conference on Pattern Recognition Applications and Methods*.

Lorenzo-Navarro, J., Castrillón-Santana, M., Sánchez-Nielsen, E., Zarco, B., Herrera, A., Martínez, I. and Gómez, M. 2021. Deep learning approach for automatic microplastics counting and classification. *Science of the Total Environment*, 765: 142728.

Luo, Y., Su, W., Xu, X., Xu, D., Wang, Z., Wu, H., Chen, B. and Wu, J. 2022. Raman Spectroscopy and Machine Learning for Microplastics Identification and Classification in Water Environments. *IEEE Journal of Selected Topics in Quantum Electronics*, 29 (4: Biophotonics): 1-8.

Mai, L., You, S.-N., He, H., Bao, L.-J., Liu, L.-Y. and Zeng, E. Y. 2019. Riverine microplastic pollution in the Pearl River Delta, China: are modeled estimates accurate? *Environmental science & technology*, 53 (20): 11810-11817.

Maximenko, N., Hafner, J. and Niiler, P. 2012. Pathways of marine debris derived from trajectories of Lagrangian drifters. *Marine pollution bulletin*, 65 (1-3): 51-62.

Meesters, J. A., Koelmans, A. A., Quik, J. T., Hendriks, A. J. and van de Meent, D. 2014. Multimedia modeling of engineered nanoparticles with SimpleBox4nano: model definition and evaluation. *Environmental science & technology*, 48 (10): 5726-5736.

Meyers, N., Catarino, A. I., Declercq, A. M., Brenan, A., Devriese, L., Vandegehuchte, M., De Witte, B., Janssen, C. and Everaert, G. 2022. Microplastic detection and identification by Nile red staining: Towards a semi-automated, cost-and time-effective technique. *Science of the Total Environment*, 823: 153441.

Michel, A. P., Morrison, A. E., Preston, V. L., Marx, C. T., Colson, B. C. and White, H. K. 2020. Rapid identification of marine plastic debris via spectroscopic techniques and machine learning classifiers. *Environmental science & technology*, 54 (17): 10630-10637.

Mountford, A. and Morales Maqueda, M. 2019. Eulerian Modeling of the three‐dimensional distribution of seven popular microplastic types in the global ocean. *Journal of Geophysical Research: Oceans*, 124 (12): 8558-8573.

Nizzetto, L., Bussi, G., Futter, M. N., Butterfield, D. and Whitehead, P. G. 2016. A theoretical assessment of microplastic transport in river catchments and their retention by soils and river sediments. *Environmental Science: Processes & Impacts*, 18 (8): 1050-1059.

Sani-Kast, N., Scheringer, M., Slomberg, D., Labille, J., Praetorius, A., Ollivier, P. and Hungerbühler, K. 2015. Addressing the complexity of water chemistry in environmental fate modeling for engineered nanoparticles. *Science of the Total Environment*, 535: 150-159.

Siegfried, M., Koelmans, A. A., Besseling, E. and Kroeze, C. 2017. Export of microplastics from land to sea. A modelling approach. *Water research*, 127: 249-257.

Unice, K., Weeber, M., Abramson, M., Reid, R., van Gils, J., Markus, A., Vethaak, A. and Panko, J. 2019. Characterizing export of land-based microplastics to the estuary-Part I: Application of integrated geospatial microplastic transport models to assess tire and road wear particles in the Seine watershed. *Science of the Total Environment*, 646: 1639-1649.

van Wijnen, J., Ragas, A. M. and Kroeze, C. 2019. Modelling global river export of microplastics to the marine environment: Sources and future trends. *Science of the Total Environment*, 673: 392-401.

Whitehead, P. G., Bussi, G., Hughes, J. M., Castro-Castellon, A. T., Norling, M. D., Jeffers, E. S., Rampley, C. P., Read, D. S. and Horton, A. A. 2021. Modelling microplastics in the river thames: Sources, sinks and policy implications. *Water*, 13 (6): 861.

Yan, X., Cao, Z., Murphy, A. and Qiao, Y. 2022. An ensemble machine learning method for microplastics identification with FTIR spectrum. *Journal of Environmental Chemical Engineering*, 10 (4): 108130.

Yu, X., Ladewig, S., Bao, S., Toline, C. A., Whitmire, S. and Chow, A. T. 2018. Occurrence and distribution of microplastics at selected coastal sites along the southeastern United States. *Science of the Total Environment*, 613: 298-305.

Yurtsever, M. and Yurtsever, U. 2019. Use of a convolutional neural network for the classification of microbeads in urban wastewater. *Chemosphere*, 216: 271-280.

Zhang, C., Wang, Q., Zhao, J., Zhao, Y. and Shan, E. 2022. Observational and model studies on transport and inventory of microplastics from a leak accident on the beaches of Yantai. *Marine pollution bulletin*, 182: 113976.
